# Human cerebrospinal fluid 6E10-immunoreactive protein species contain amyloid precursor protein fragments

**DOI:** 10.1371/journal.pone.0212815

**Published:** 2019-02-28

**Authors:** Marianne K. O. Grant, Maureen Handoko, Malgorzata Rozga, Gunnar Brinkmalm, Erik Portelius, Kaj Blennow, Karen H. Ashe, Kathleen R. Zahs, Peng Liu

**Affiliations:** 1 Department of Neurology, University of Minnesota, Minneapolis, Minnesota, United States of America; 2 N. Bud Grossman Center for Memory Research and Care, University of Minnesota, Minneapolis, Minnesota, United States of America; 3 Institute of Neuroscience and Physiology, Department of Psychiatry and Neurochemistry, Sahlgrenska Academy at University of Gothenburg, Mölndal, Sweden; 4 Clinical Neurochemistry Laboratory, Sahlgrenska University Hospital, Mölndal, Sweden; 5 Department of Neuroscience, University of Minnesota, Minneapolis, Minnesota, United States of America; 6 Geriatric Research, Education, and Clinical Centers, Veterans Affairs Medical Center, Minneapolis, Minnesota, United States of America; Nathan S Kline Institute, UNITED STATES

## Abstract

In a previous study, we reported that levels of two types of protein species—a type of ~55-kDa species and a type of ~15-kDa species—are elevated in the lumbar cerebrospinal fluid (CSF) of cognitively intact elderly individuals who are at risk for Alzheimer’s disease (AD). These species are immunoreactive to the monoclonal antibody 6E10, which is directed against amino acids 6–10 of amyloid-β (Aβ), and their levels correlate with levels of total tau and tau phosphorylated at Thr181. In this study, we investigated the molecular composition of these AD-related proteins using immunoprecipitation (IP)/Western blotting coupled with IP/mass spectrometry. We show that canonical Aβ_1-40/42_ peptides, together with amyloid-β precursor protein (APP) fragments located N-terminally of Aβ, are present in the ~55-kDa, 6E10-immunoreactive species. We demonstrate that APP fragments located N-terminally of Aβ, plus the N-terminal region of Aβ, are present in the ~15-kDa, 6E10-immunoreactive species. These findings add to the catalog of AD-related Aβ/APP species found in CSF and should motivate further study to determine whether these species may serve as biomarkers of disease progression.

## Introduction

Alzheimer’s disease (AD) is a progressive neurodegenerative disorder that is pathologically characterized by extracellular amyloid plaques, intracellular neurofibrillary tangles and neuronal loss in selected brain regions. Amyloid-β (Aβ) peptides, the main component of plaques, are generated from sequential processing of amyloid precursor protein (APP) by β- and γ- secretase with Aβ_1–40_ being most abundant, while Aβ peptides ending at position 42 (Aβ_42_) are most hydrophobic and the major component of amyloid plaques [1]. The 1992 finding that Aβ peptides could be secreted to the cerebrospinal fluid (CSF) [2] stimulated more than two decades of study to determine whether CSF Aβ could serve as a biomarker to diagnose AD or monitor disease progression—research that is still ongoing. Using enzyme-linked immunosorbent assay (ELISA) to measure Aβ_42_, Motter et al. showed a significant reduction in peptide levels in CSF of AD patients [3], which has been reproduced later in multiple studies (for review, see for example [4, 5]). Aβ_40_, the most abundant Aβ variant present in CSF [6], shows similar levels in AD patients and age-matched cognitively normal individuals [7]. Since levels of Aβ_40_ can be used to balance inter-individual variations in total Aβ production, the use of the Aβ_42_/Aβ_40_ ratio has been reported to improve diagnostic accuracy [8, 9]; however, the reduction in CSF Aβ_42_ also occurs in patients with neurodegenerative disorders other than AD [10–12]. Although combining CSF Aβ biomarkers with amyloid neuroimaging biomarkers (*e*.*g*., PiB amyloid positron emission tomography) and other CSF biomarkers (*e*.*g*., total tau (t-tau) and phosphorylated tau (p-tau)) has proven to be useful for AD diagnosis, other molecular biomarkers are needed to enhance the sensitivity and specificity of diagnosis of various neurodegenerative disorders as well as to differentiate clinical phenotypes of AD.

There is a developing consensus that neurological dysfunction in AD is more closely associated with the presence Aβ oligomers than with plaques (for reviews, see for example [13, 14]). A growing body of evidence shows the presence of Aβ oligomers in human brains and biological fluids (for reviews, see for example [15–17]), suggesting that Aβ oligomers in human biofluids may serve as AD biomarkers not only for clinical diagnosis but also for use as tools for increased understanding of disease pathogenesis. Various methodologies have been used for quantitative analysis of Aβ oligomers in CSF, including but not limited to ELISA, fluorescence correlation spectroscopy, bio barcode assay, protein misfolding cyclic amplification assay, fluorescence resonance energy transfer/flow cytometry-based assays, surface fluorescence intensity distribution analysis, immunoprecipitation (IP)/Western blotting (WB), and ultrasensitive bead-based immunoassays (for review, see for example [18]). Whether levels of Aβ oligomers differ between AD patients and cognitively normal individuals remains debated [19–29]. Inconsistent findings might be attributed to the low abundance, heterogeneity and instability of Aβ oligomers in CSF, and possibly interfering signals from monomeric Aβ; in addition, there is a potential for misinterpreting the results of assays that employ antibodies that are not selective for Aβ.

In a previous study of human lumbar CSF, we identified two distinct types of protein species that are immunoreactive to the monoclonal antibody 6E10, directed against amino acids 6–10 of Aβ, which we tentatively identified as Aβ oligomers [23]. The CSF levels of both 6E10-reactive species are age-dependently elevated, and their levels are significantly increased in cognitively normal elderly individuals at risk for AD. Interestingly, the CSF levels of these 6E10-reactive species also correlate with levels of t-tau and p-tau at Thr181 [23], suggesting the relevance of these species to AD. Since 6E10 also recognizes full-length APP and APP metabolites other than Aβ [30], it is conceivable that the two types of CSF species may not be comprised solely of full-length Aβ peptides. As such, the actual identities of these species, previously deemed oligomeric Aβ [23], are questionable. Indeed, the presence of various APP metabolites in lumbar CSF has been well documented [31–34]. In addition, a recent study suggests that non-Aβ proteins appear to be part of soluble Aβ aggregates in human specimens [35]. Taken together, these findings indicate that the 6E10-immunoreactive proteins in human lumbar CSF are comprised of various APP metabolites besides Aβ.

In this study, we test the hypothesis that the two types of AD-relevant, 6E10-immunoreactive CSF protein species contain APP fragments other than full-length Aβ_1-40/42_ peptides. Using (IP/WB) coupled with IP/mass spectrometry (MS), we show that APP fragments located N-terminally of Aβ, in addition to canonical Aβ_1-40/42_, are present in the ~55-kDa species. We also show that APP fragments located N-terminally of Aβ plus the N-terminal region of Aβ are present in the ~15-kDa species.

The findings of this study refine our understanding of the nature of CSF protein species previously shown to be elevated in individuals at risk for AD [23]. Characterization of the disease-associated molecules that are present in biological specimens may provide insight into underlying disease processes and inform effective AD prevention and therapy.

## Materials and methods

### Lumbar CSF collection and handling

CSF samples (S1 Table) were obtained from the Clinical Neurochemistry Laboratory, Sahlgrenska University Hospital, Sweden. The CSF samples were de-identified left-over aliquots from clinical routine analyses, following a procedure approved by the Ethics Committee at University of Gothenburg (EPN 140811). After these de-identifying procedures, according to the Swedish Biobank law (Biobanks in Medical Care Act) and The Act concerning the Ethical Review of Research Involving Humans, these samples are no longer covered by human ethics regulations, and they can be used in method development, comparisons, or validation studies. All samples had normal basic (cell count, albumin ratio, immunoglobulin G (IgG) index) biomarker values, and were selected based on core (t-tau, p-tau and Aβ_42_) CSF biomarkers levels. Samples with an “AD” amyloid biomarker profile had low CSF Aβ_42_ (<400 pg/mL) while samples with a normal amyloid profile had CSF Aβ_42_ levels >550 pg/mL. The subjects providing CSF with a normal amyloid profile had minor psychiatric or neurological symptoms, but were judged, based on basic CSF analyses and core (Abeta42, t-tau and p-tau) AD biomarker levels [36] not to have any disorders characterized by blood-brain barrier damage, neuroinflammation, neurodegeneration or AD pathology. CSF was collected by lumbar puncture in polypropylene tubes, centrifuged at room temperature, 2,000 x *g* for 10 min, and stored at -80°C prior to shipment to Minnesota, USA. Upon arrival, samples were thawed at room temperature in the presence of protease and phosphatase inhibitors (1 mM phenylmethylsulfonyl fluoride, 2 mM 1,10-phenanthroline monohydrate, and 1× protease inhibitor cocktail (Sigma-Aldrich, St. Louis, MO), phosphatase inhibitor cocktail A (Santa Cruz Biotechnology, Santa Cruz, CA) and phosphatase inhibitor cocktail 2 (Sigma-Aldrich); for each protease or phosphatase inhibitor cocktail, the volume ratio of inhibitor:CSF was 1:1,000), aliquoted, and re-frozen. Samples therefore went through two freeze-thaw cycles prior to biochemical analyses. All procedures were approved by the Institutional Review Board at the University of Minnesota.

S2 Table lists the amyloid status of the CSF samples used in this study.

### Immunoprecipitation and Western blotting (IP/WB)

**Fig 1:**
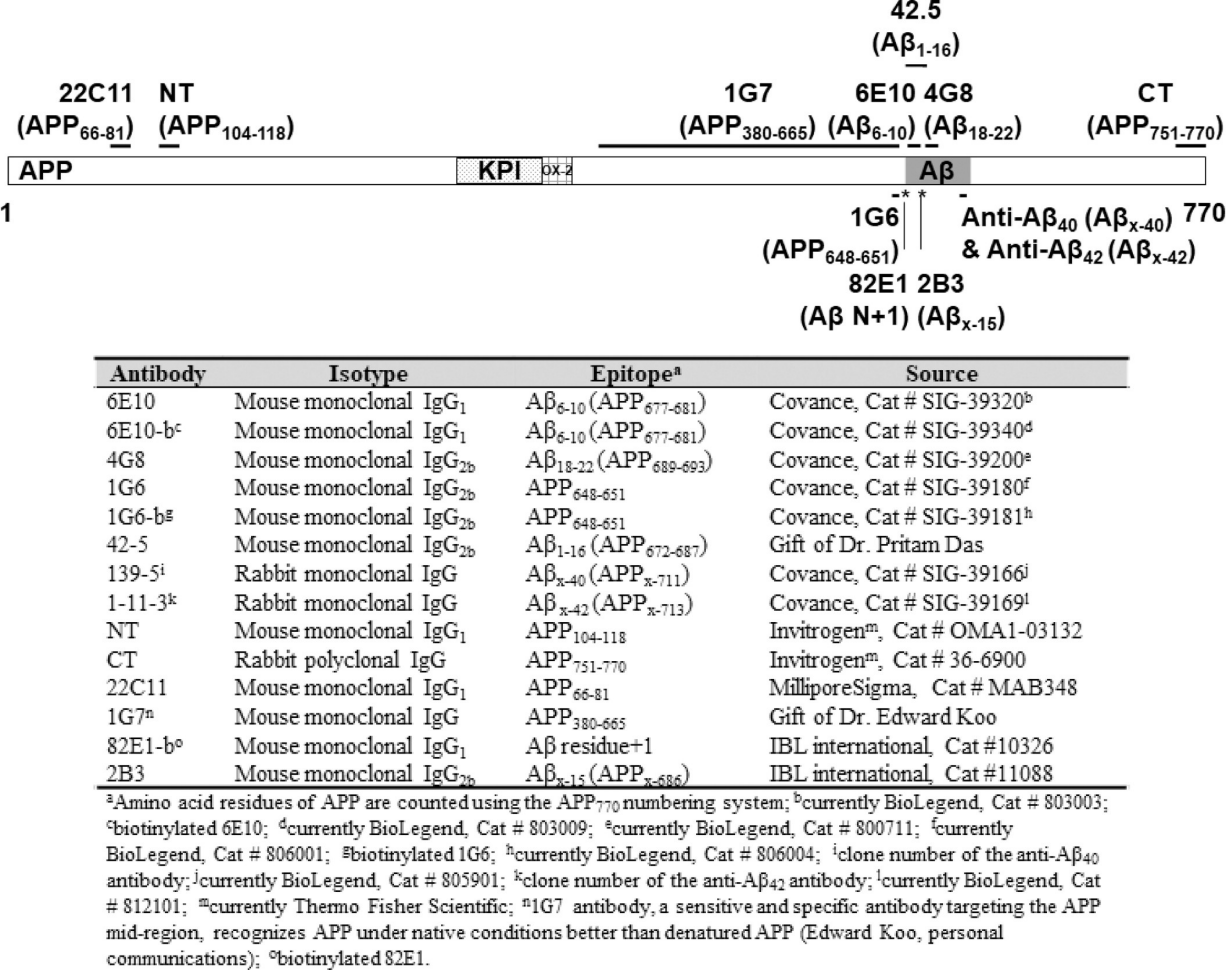
Anti-Aβ/APP antibodies used in this study. The epitopes targeted by antibodies are labeled in the upper panel. The APP_695_ isoform lacks the Kunitz-like protease inhibitor (KPI) domain and the OX-2 antigen domain, and the APP_751_ isoform lacks only the OX-2 domain. Detailed information of these antibodies are shown in the lower panel.

Aβ/APP metabolites were immunoprecipitated from 160–500 μL of CSF using a series of antibodies (**Fig 1**). CSF samples were thawed and brought to a volume of 500 μL through the addition of immunoprecipitation dilution buffer (IPDB: 50 mM Tris-HCl, 150 mM NaCl, pH 7.4) containing the protease and phosphatase inhibitors listed in Section **Lumbar CSF collection and handling**. The volume ratio of inhibitor stock solution to IPDB-diluted CSF sample was 1:1,000. Samples were first depleted of endogenous IgGs with Sepharose Fast Flow Protein G beads (GE Healthcare, Piscataway, NJ; 50 μL of slurry per 500 μL of sample). Samples were then incubated overnight at 4 ^o^C with capture antibody (5 μg per reaction for 6E10, 8 μg per reaction for other antibodies) and Protein G-coated magnetic beads (Dynabeads, Life Technologies, Grand Island, NY; 50 μL of slurry per reaction), washed for 20 min at 4 ^o^C with immunoprecipitation buffer A (IP Buffer A: 50 mM Tris-HCl, 300 mM NaCl, 1 mM ethylenediaminetetraacetic acid (EDTA), 0.1% (v/v) Triton X-100, pH 7.4), then for 20 min at 4 ^o^C with immunoprecipitation buffer B (IP Buffer B: 50 mM Tris-HCl, 150 mM NaCl, 1 mM EDTA, 0.1% (v/v) Triton X-100, pH 7.4). Proteins were eluted by boiling the magnetic beads at 95°C in 30 μL of sodium dodecyl sulfate (SDS)-polyacrylamide gel electrophoresis (PAGE) loading buffer (500 mM Tris-HCl, 24% (v/v) glycerol, 8% (w/v) SDS, 0.01% (w/v) Coomassie brilliant blue, 0.1% (v/v) phenol red, 710 mM β-mercaptoethanol, pH 8.0); elutates were size-fractionated on 10.5–14% Tris-HCl or 10–20% Tris-Tricine precast gels (Bio-Rad, Hercules, CA), and electrophoretically transferred onto 0.2-μm nitrocellulose membranes at a constant current of 0.4 A for 3 hr at 4°C. Membranes were boiled twice in phosphate buffered saline (PBS) for 25 sec and 15 sec, with a 4-min interval between periods of boiling. Membranes were then blocked with blocking buffer (5% (w/v) bovine serum albumin (Sigma-Aldrich) in TBS-T (Tris Buffered Saline with 0.1% (v/v) Tween 20)) for 1hr at room temperature, and incubated overnight at 4 ^o^C with biotinylated 6E10 (BioLegend, San Diego, CA; 1:2,500 = 0.4 μg/mL blocking buffer), biotinylated 82E1 (IBL international, Hamburg, Germany; 1:1,000 = 0.1 μg/mL blocking buffer), or biotinylated 2B3 (IBL international, 1:2,000 = 0.9 μg/mL blocking buffer). Following 5 X 5-min washes in TBS-T, membranes were incubated for 10 min at room temperature in NeutrAvidin-horseradish peroxidase (HRP) (Life Technologies, Carlsbad, CA; 1:5,000), washed again (5 X 5-min in TBS-T), then developed using the West Pico electrochemiluminescence detection system (Thermo Scientific, Rockford, IL).

For direct WB of lumbar CSF, 40 μL of samples were mixed with 13 μL of SDS-PAGE loading buffer, boiled at 95°C, and size-fractionated on 10.5–14% Tris-HCl precast gels (Bio-Rad). WB was performed following the procedures described above. Specifically, membranes were incubated overnight at 4 ^o^C with primary monoclonal antibodies biotinylated 6E10 (BioLegend, 1:2,500 = 0.4 μg/mL blocking buffer), 42.5 (a kind gift of Dr. Pritam Das, 1:2,500 = 0.67 μg/mL blocking buffer), 4G8 (BioLegend, 1:2,500 = 0.4 μg/mL blocking buffer), anti-Aβ_x-40_ (BioLegend, 1:5,000 = 0.2 μg/mL blocking buffer), anti-Aβ_x-42_ (BioLegend, 1:5,000 = 0.1 μg/mL blocking buffer), biotinylated 1G6 (BioLegend, 1:2,500 = 0.4 μg/mL blocking buffer), 1G7 (a kind gift of Dr. Edward Koo, 1:2,500 = 0.4 μg/mL blocking buffer), 22C11 (MilliporeSigma, Burlington, MA; 1:2,500 = 0.4 μg/mL blocking buffer), and antibody targeting C-terminal APP (CT) (Thermo Fisher Scientific, Waltham, MA; 1:2,500 = 0.1 μg/mL blocking buffer). Following multiple washes (5 X 5-min in TBS-T), membranes were incubated in NeutrAvidin-HRP for biotinylated antibodies, biotinylated goat-anti-mouse IgG (msIgG) (Thermo Fisher Scientific, 1:60,000) at room temperature for 1 hr for antibodies 42.5, 4G8, 1G7 and 22C11, or biotinylated donkey-anti-rabbit IgG (rbtIgG) (Jackson ImmunoResearch Laboratories Inc., West Grove, PA, 1:50,000) at room temperature for 1 hr for antibodies anti-Aβ_x-40_, anti-Aβ_x-42_ and CT. Following multiple washes (5 X 5-min in TBS-T), blots were either directly developed (for membranes incubated with biotinylated primary antibodies) using the West Pico or the West Femto Maximum Sensitivity electrochemiluminescence detection system (Thermo Scientific) or incubated in NeutrAvidin-HRP (for membranes incubated with biotinylated secondary antibodies) prior to development. As negative controls, membranes were also probed using msIgG (MilliporeSigma, 1: 2,500 = 0.4 μg/mL blocking buffer), rbtIgG (MilliporeSigma, 1: 2,500 = 0.4 μg/mL blocking buffer) or NeutrAvidin-HRP.

### Size exclusion chromatography (SEC)

Five hundred μL of CSF from a normal individual was depleted of endogenous IgGs and loaded onto a Superdex 200 10/300 GL column (GE Healthcare) and eluted with 50 mM ammonium acetate, pH 8.5 at a flow rate of ~0.3 mL/min. Fractions of 250 μL were collected, and 125 μL aliquots of each selected fraction were analyzed by WB. Specifically, proteins were separated in 10–20% Precast Tris-Tricine gels (Bio-Rad), electrophoretically transferred onto nitrocellulose membranes, and probed using biotinylated 6E10 or anti-Aβ_x-40/42_.

### Immunodepletion

Prior to immunoprecipitation by 6E10, CSF was depleted of various APP metabolites using antibodies 6E10, 4G8, 1G6 and CT, or msIgG (negative control). To avoid retaining any antibodies in the CSF-containing supernatant after immunodepletion, and thus affecting signal detection in the following immunoprecipitation step, immunodepletion antibodies were first covalently crosslinked to the matrix. This section contains two subsections. The first subsection describes the methods of immobilizing antibodies onto Protein G magnetic beads, and the second subsection describes how immunodepletion was performed.

#### 1) Antibody immobilization on Protein G magnetic beads

Monoclonal antibodies 6E10, 4G8 and 1G6; msIgG; and polyclonal antibody CT were covalently linked to Protein G-coupled magnetic beads (Dynabeads, Life Technologies; 200 μg of antibody per 1 mL of Dynabead slurry) using dimethyl pimelimidate (Thermo Fisher Scientific) as the crosslinker, according to the manufacturer’s instructions. Prior to use, beads were pre-treated to wash off any antibody that was not covalently linked. Specifically, beads were washed for 15 min at 4 ^o^C with IP Buffer A, then 15 min at 4 ^o^C with IP Buffer B, followed by 5 min at 4 ^o^C with IP Buffer B with 1% (v/v) Trtion X-100. To monitor any shedding of antibody from the beads, the beads were incubated and mixed in 30 μL of elution buffer (100 mM glycine-HCl, 1% (w/v) *n*-Octyl *β*-D-thioglucopyranoside (OTG), pH 2.8) for 3 min at room temperature. The eluate was collected, and 10 μL of SDS-PAGE loading buffer was added to the eluate. The sample was boiled at 95°C for 5 min, and loaded onto a 10–20% Tris-Tricine precast gel. Gel electrophoresis and WB were performed as described above, except that goat-anti-msIgG-conjugated to HRP (Jackson ImmunoResearch Laboratories Inc.) or goat-anti-rbt IgG-conjugated to HRP (for CT) (Jackson ImmunoResearch Laboratories Inc.) was used to probe blots.

#### 2) Immunodepletion

Immunodepletion antibodies or msIgG were separately immobilized on Protein G-coated magnetic beads (Dynabeads, Life Technologies) (100 μg antibody/500 μL slurry) using the protocol described in Subsection **Antibody immobilization on Protein G magnetic beads**. Immobilized antibodies were then incubated with 250 μL of CSF samples overnight at 4 ^o^C. The supernatant was then collected and incubated with 6E10 according to the protocol described in Section **Immunoprecipitation and Western blotting (IP/WB)**.

### Preparation of samples for mass spectrometry

CSF samples were subjected to immunoprecipitation and SDS-PAGE at the University of Minnesota, and gel pieces were returned to the University of Gothenburg for mass spectrometric analysis. In order to avoid any possible interference from commonly-used protein stains, these pieces were excised from unstained gels. This section is comprised of two parts. The first subsection (‘Pilot studies”) describes experiments we conducted to determine the feasibility of precisely identifying regions of interest on unstained gels, and the second subsection (“Sample preparation”) describes how the ~55- and the ~15- kDa, 6E10-reactive proteins were isolated in gel pieces from CSF specimens.

#### 1) Pilot studies

A series of pilot experiments were first conducted to determine whether it was possible to accurately localize regions of interest on unstained gel by referring to WB processed in parallel (**S1 Fig**). Specifically, a volume of each sample was allocated to a “reference” lane on the gel, and the remaining volume was divided into “analytic” lanes. The reference lane and the set of analytic lanes were flanked by lanes containing pre-stained molecular weight standards (Bio-Rad). After separating the proteins by electrophoresis, the reference lane (with flanking lanes) was cut off and subjected to a full WB procedure, during which time the analytic lanes were stored in a moist 4°C chamber. Finally, the analytic lanes were overlaid on the film record of the reference lane WB using the molecular weight standards for alignment, and the pieces of unstained gel overlaying the bands of interest were excised.

We first determined that the blotting process did not distort the positions of molecular weight protein standards. Pre-stained molecular weight protein standards (Bio-Rad) were separated by SDS-PAGE (10–20% Tris-Tricine or 10.5–14% Tris-HCl precast gels, Bio-Rad), and the positions of the protein standards were recorded. The protein standards were then electrophoretically transferred to a nitrocellulose membrane, which was then boiled for epitope retrieval. The positions of the stained protein standards on the membrane matched those recorded prior to transfer.

Because the WB procedure takes approximately 20 hr following the SDS-PAGE step, we next determined that proteins largely remained in place in unfixed gels stored for that length of time. Protein samples included synthetic Aβ_40_ and Aβ_42_ as well as 6E10-immunoreactive species that were immunoprecipitated from a sample of cadaveric ventricular CSF (obtained from Geriatric Research, Education, and Clinical Centers, Veterans Affairs Medical Center, Minneapolis, MN; the provider is a male AD patient who died at the age of 84 years old, and the post-mortem interval of sample harvest is 4 hr), according to the protocol described in Section **Immunoprecipitation and Western blotting (IP/WB)**. (Briefly, CSF was incubated with 6E10 that was covalently linked to Protein G-coupled magnetic beads, and immunocaptured species were then eluted using 0.5% (w/v) OTG in 100 mM glycine, pH 2.8.) Duplicate sets of protein samples were loaded onto each half of a 10–20% Tris-Tricine precast gel (Bio-Rad), and proteins were separated by electrophoresis. One half of the gel was then immediately subjected to the remainder of the WB protocol (Section **Immunoprecipitation and Western blotting (IP/WB)**), while the other half was left on the back of the gel cassette and stored for 20 hr in a moist 4°C chamber before continuing with the WB protocol. Qualitatively, the blots processed immediately and after gel storage appeared quite similar.

#### 2) Sample preparation

**Fig 2:**
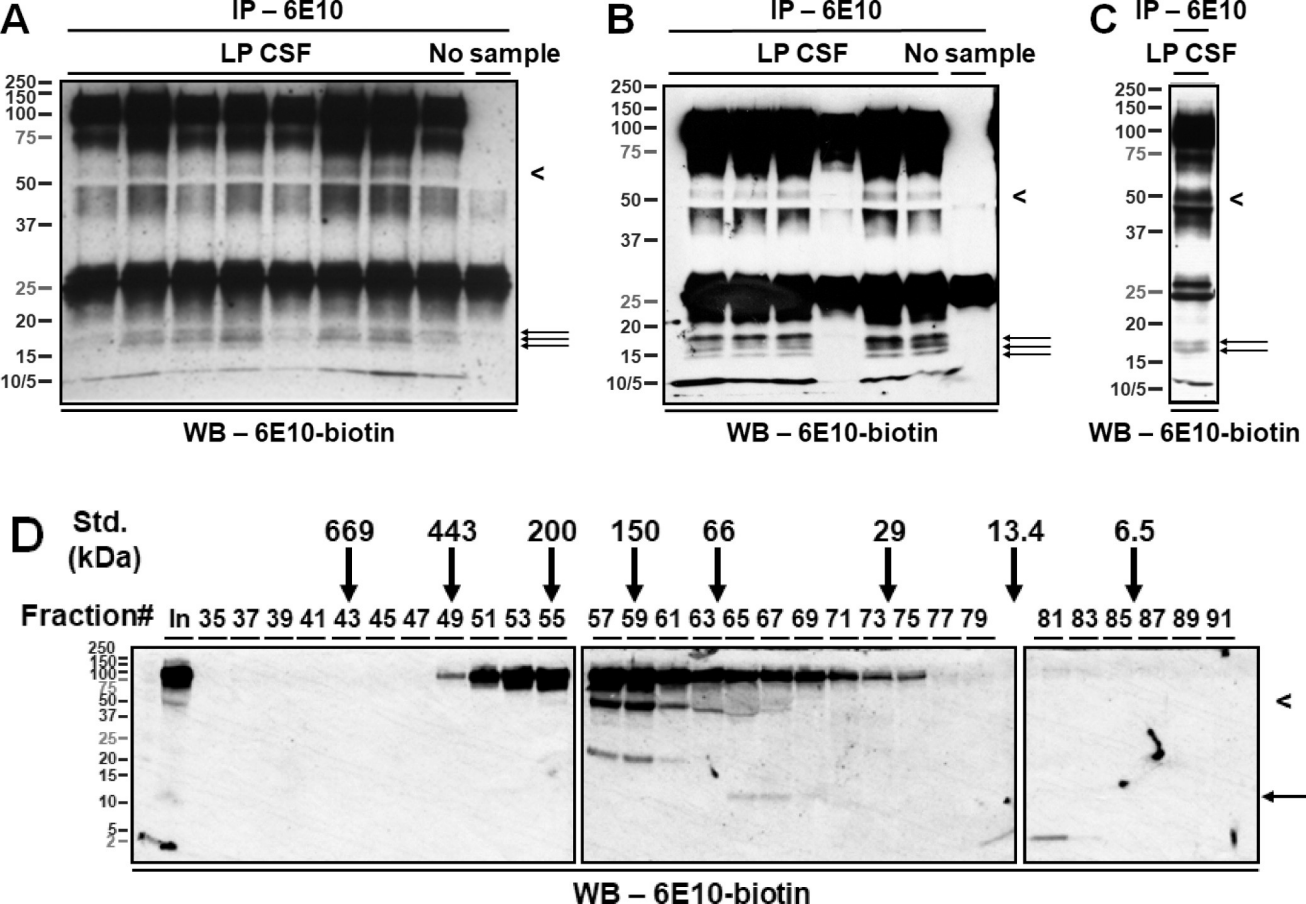
6E10-immunoreactive proteins in human CSF. (**A-C**) Representative IP/WB. Monoclonal antibody 6E10 was used to immunoprecipitate Aβ/APP metabolites from human CSF samples (250 μL per sample). Immunocaptured proteins were separated by SDS-PAGE, and detected by biotinylated 6E10. Note the proteins migrating at ~55 kDa (arrowhead) and at ~15 kDa (arrows), which are further characterized in this study. Proteins migrating at ~100 kDa and at <10 kDa likely represent the soluble, α-secretase-cleaved APP metabolites (sAPPα) and monomeric canonical Aβ_1-40/42_, respectively. Proteins migrating at ~70 kDa likely represent APP metabolites whose identities are beyond the scope of investigation of this study. Three sets of samples (A, 250 μL of CSF per lane; B, 240 μL of CSF per lane; C, 160 μL of CSF) were immunoprecipitated by monoclonal antibody 6E10; the eluates were separated by SDS-PAGE using 10.5–14% Tris-HCl gels and detected by biotinylated 6E10. The IP/WB experiments were performed by different experimenters at three different times spanning a five-year period; nonetheless, Fig 2A, 2B and 2C show a similar WB pattern. (**D**) WB showing fractionation of 6E10-immunoreactive species by SEC. The ~100- and ~70- kDa proteins were eluted together in fractions 49–75, the ~55-kDa proteins in fractions 57–63, the ~15-kDa proteins in fractions 65–69, and the <10-kDa proteins in fractions 79–83. No <10-kDa proteins were detected in the early fractions containing the ~55- and the ~15- kDa species, and no ~55 kDa or ~15 kDa species were detected in the late fractions containing the <10-kDa species. Five percent of the volume of the injected CSF sample is shown in the first lane (In). Vertical arrows indicate the fractions in which globular protein standards of the indicated molecular weights were eluted. The mismatch between the predicted elution fraction and molecular weights estimated by SDS-PAGE suggests that the 6E10-immunoreactive Aβ/APP metabolites do not migrate through the column as globular proteins. LP CSF, CSF obtained by lumbar puncture from living providers.

Eight CSF samples from individual patients were screened for the presence of a ~55- and a ~15- kDa, 6E10-immunoreactive species (**Fig 2A**). The four samples (Lanes #2, 4, 6 and 7, **Fig 2A**) with the highest levels of the ~55-kDa species were pooled for subsequent analysis.

The pooled CSF was first immunodepleted using Sepharose Fast Flow Protein G beads, as described in Section **Immunoprecipitation and Western blotting (IP/WB)**. Immunodepleted CSF was then incubated overnight at 4°C with an immunoaffinity matrix composed of either monoclonal antibody 6E10 or msIgG (as a control) that was covalently linked to Protein G-coupled magnetic beads, prepared as described in Subsection **1) Antibody immobilization on Protein G magnetic beads** (approximately 1 mL of CSF per 300 μL of beads). Following this overnight incubation, the beads were washed twice with IPDB containing 0.1% (w/v) OTG and twice with IPDB containing 1% (w/v) OTG. Proteins were then eluted (0.5% (w/v) OTG in 100 mM glycine, pH 2.8; 35 μL of buffer per 300 μL of beads, 1-min incubation with mixing by tapping tube; three rounds). Eluates from the three rounds were pooled and concentrated to ~2.5X in a vacufuge. For this study, we conducted four rounds of sample preparation. For each round, approximately 3 mL of CSF was applied to the 6E10 immunoaffinity matrix, and 2.6 mL to the msIgG (control) matrix.

Immunocaptured proteins were then separated by SDS-PAGE, using either 10.5–14% Tris-HCl or 10–20% Tris-Tricine precast gels (Bio-Rad), to achieve better isolation of the ~55- and the ~15- kDa species, respectively. Two gels (of the same type) were run for each round of sample preparation. The concentrated eluates from the 6E10 immunoaffinity capture were divided into 4 X 25.7 μL volumes for loading into analytic lanes (each analytic lane thus contained the proteins captured from ~664 μL of CSF), and 2 X 8.7 μL volumes for loading into reference lanes (each lane containing the proteins captured from ~225 μL of CSF); the concentrated eluates from the control msIgG matrix were apportioned into four analytic lanes (each lane containing the proteins captured from ~664 μL of CSF). Each sample was brought to a volume of 30 μL by the addition of 2.5X elution buffer (1.25% (w/v) OTG in 250 mM glycine, pH 2.8), and 10 μL of SDS-PAGE loading buffer and 1.875 μL of 5 M NaCl were added (to achieve a final concentration ~300 mM). For each marker lane, 8 μL of pre-stained protein standards were added to 22 μL of 2.5X elution buffer, 7 μL of SDS-PAGE loading buffer and 1.875 μL of 5 M NaCl. Prior to loading the gel, all mixtures were boiled for 7 min with agitation. Gel lanes were ordered as follows: 1) standards, 2–3) eluate from msIgG matrix (analytic), 4–5) standards, 6) eluate from 6E10 matrix (reference), 7–8) standards, 9–10) eluate from 6E10 matrix (analytic), 11) standards, 12) blank (30 μL of 2.5X elution buffer, 10 μL of loading buffer, 1.875 μL of 5 M NaCl). Gels were run at 80–90 V, until the dye front reached the bottom of the gel.

Following SDS-PAGE, the reference lane and two flanking marker lanes were subjected to the WB protocol described in Section **Immunoprecipitation and Western blotting (IP/WB)**, using biotinylated 6E10 as the detection antibody. The locations where proteins of interest potentially migrated were identified using the procedure described in Subsection **1) Pilot studies**, and the corresponding gel pieces were excised from the analytic lanes.

Gel pieces were washed three times in 50 mM ammonium bicarbonate (pH 7.8), covered with acetonitrile, and vacufuged until dry. Dried gel pieces were stored at room temperature prior to shipping to Sweden (at ambient temperature).

### Peptide liquid chromatography fractionation and mass spectrometry

To analyze CSF samples, a Q Exactive mass spectrometer (Thermo Fisher Scientific) coupled to a Dionex Ultimate 3000 nanoflow LC system (Thermo Fisher Scientific) was used. This section is comprised of four parts. The first subsection describes the methods for in-gel trypsin digestion of protein and peptide extraction, the second subsection describes peptide separation using liquid chromatography, the third subsection describes parameters used for mass spectrometry analysis, and the fourth subsection describes the methods for database search to determine peptide identity.

#### 1) In-gel trypsin digestion and peptide extraction

Gel processing and in-gel digestion were performed in a keratin-free laminar flow cabinet in order to prevent the risk of sample keratin contamination. All the working solutions were prepared immediately before use. First, gel slices were dehydrated in 200 μL of 100% (v/v) acetonitrile for 10 min at room temperature. To ensure complete dehydration, this step was repeated once. After removal of acetonitrile, the samples were left at room temperature for 10 min in a laminar chamber to dry completely. Dried gel slices were incubated in 100 μL of reduction solution (25 mM ammonium bicarbonate, 10 mM dithiothreitol, pH 7.8) at 56°C in a heating block for 30 min. After cooling, the reduction solution was removed and gel slices were incubated with 100 μL of alkylation solution (25 mM ammonium bicarbonate, 55 mM iodoacetamide, pH 7.8) for 20 min in the dark at room temperature. After discarding the alkylation solution, samples were washed in 100 μL of 50 mM ammonium bicarbonate, pH 7.8 for 10 min at room temperature. Supernatant was removed and gel slices were dehydrated in 100 μL of acetonitrile for 5 min at room temperature. After removal of the supernatant, this dehydration step was repeated twice, and samples were then dried in a laminar chamber for 10 min at room temperature. Dried gel slices were then covered with trypsin solution (12.5 ng/μL in 25 mM ammonium bicarbonate, pH 7.8). Digestion proceeded overnight at 37°C in a heating block. After digestion, peptides were extracted from the gel cubes with 40 μL of 5% (v/v) formic acid by incubation while shaking at 37°C for 15 min. Finally, supernatants were transferred to siliconized Eppendorf tubes and dried down using a SpeedVac concentrator. Dried samples were stored at -80°C pending analysis.

#### 2) Liquid chromatography separation of peptides

For liquid chromatography separation of peptides, the dried samples were reconstituted in 7 μL of 2% (v/v) acetonitrile/0.05% (v/v) trifluoroacetic acid in water. Six μL of the reconstituted samples were loaded onto an Acclaim PepMap C18 trap column (length: 20 mm, i.d.: 75 μm, particle size: 3 μm, pore size 100 Å, Thermo Fisher Scientific) at a flow rate of 5 μL/min, and desalted using the same solvent (2% (v/v) acetonitrile/0.05% (v/v) trifluoroacetic acid in water). Samples were then separated on a reversed-phase Acclaim PepMap C18 analytical column (length: 150 mm, i.d.: 75 μm, particle size: 2 μm, pore size: 100 Å, Thermo Fisher Scientific) using a 50-min gradient from 3% acetonitrile (v/v)/0.1% (v/v) formic acid to 40% (v/v) acetonitrile/0.1% (v/v) formic acid in water at a flow rate of 300 nL/min.

#### 3) Mass spectrometry

For the Q Exactive MS analysis, acquisition parameters were as follows: Spray voltage, +1.7 kV; capillary temperature, 250°C; MS1 scan range, *m/z* 350–1,400; resolution setting 70,000 at *m/z* 200; AGC target 10^6^; maximum injection time 250 msec; and no lock mass was used. The instrument was operated in *data dependent* mode selecting the top ten peaks with charge states, 2+, 3+, or 4+ for fragmentation (MS2) using so-called higher-energy collision induced dissociation (HCD) as follows: selection threshold, 1.7 × 10^4^; isolation width, *m/z* 2.0; normalized collision energy, 28%; resolution setting 17,500; AGC target 5 × 10^4^; maximum injection time 60 msec; and dynamic exclusion 5 sec.

#### 4) Database search

Database searches were performed with Proteome Discoverer v2.1 (Thermo Fisher Scientific) using the Mascot search engine v2.6.1. Spectra were searched against both the regular Swiss-Prot and its isoform database. Search parameters were as follows: fragment types, CID/HCD; enzyme, semiTrypsin; maximum missed cleavage sites, 2; fragment mass tolerance, 50 mmu; precursor mass tolerance, 10 ppm; dynamic modifications, oxidation (M); static modifications, carbamidomethylation (C). Since the sample complexity was low, the fixed value peptide spectrum match validator was used.

## Results

### Immunoprecipitation/Western blotting revealed 6E10-immunoreactive APP metabolites in the ~55- and the ~15- kDa species from lumbar CSF

Using Immunoprecipitation/Western Blotting (IP/WB) with 6E10 as both the capture and detection antibody, we identified ~100-, ~70-, ~55-, ~15- and <10- kDa proteins in lumbar CSF of elderly individuals (**Fig 2A–2C**). The ~55- and the ~15- kDa proteins correspond to the two species whose levels have previously been shown to be age-dependently elevated [23]. To confirm that the ~55- and the ~15- kDa, 6E10-immunoreactive proteins do not arise from SDS-induced aggregation of endogenous CSF Aβ, we fractionated CSF proteins under non-denaturing conditions using SEC. Upon WB analysis of individual fractions using biotinylated 6E10, we showed that the ~55- and the ~15- kDa proteins were eluted in different fractions than the <10-kDa proteins, presumably comprised of monomeric Aβ. These results indicate that the ~55- and the ~15- kDa, 6E10-immunoractive proteins are naturally-occurring proteins in lumbar CSF (**Fig 2D**).

**Fig 3:**
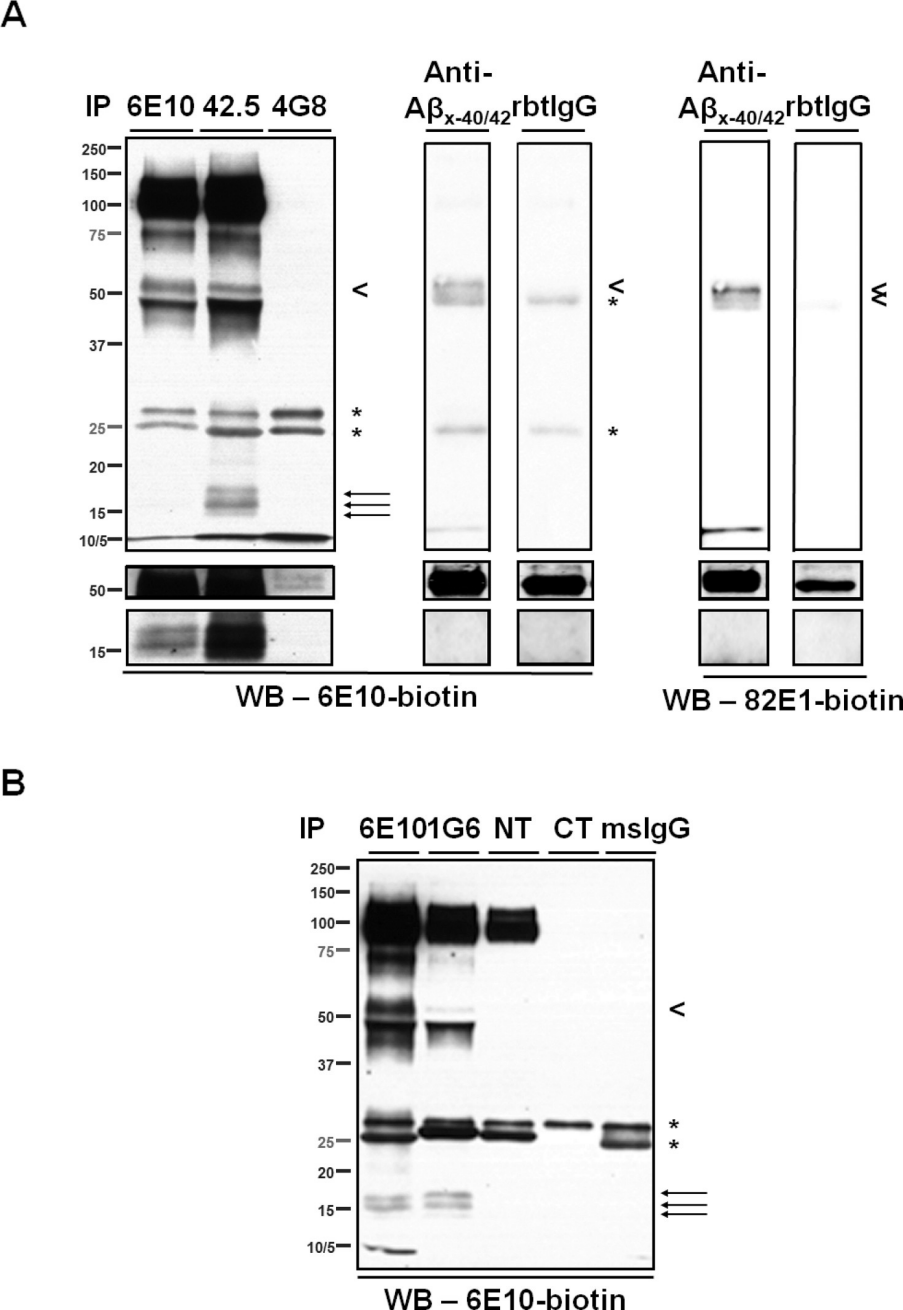
Immunological characterization of the ~55- and the ~15- kDa, 6E10-immunoreactive proteins in CSF. (**A**) A series of antibodies directed against various epitopes of Aβ were used to immunoprecipitate target proteins from CSF, and blots were probed with biotinylated- 6E10 or 82E1. Capture antibodies are indicated by the labels above each lane. Top panels show short exposure (30 sec), and bottom panels show longer exposure (3 min). Note that the ~55-kDa proteins (arrowheads) were immunoprecipitated by monoclonal antibodies targeting the N-terminal region (6E10 and 42–5), mid-region (4G8) and the C-terminal end of Aβ (anti-Aβ_x-40/42_); whereas the ~15-kDa proteins (arrows) were only immunoprecipitated by monoclonal antibodies targeting the N-terminal region of Aβ. (**B)** A series of antibodies directed against various epitopes of APP were used to immunoprecipitate target proteins from CSF, and blots were probed with biotinylated 6E10. Capture antibodies are indicated by the labels above each lane. Both the ~55- and the ~15- kDa species were immunoprecipitated by monoclonal antibody 1G6 directed against an APP epitope N-terminally adjacent to Aβ, but not by antibodies directed against the N- (“NT”) or C- (“CT”) termini of APP. Mouse and rabbit IgGs (msIgG, rbtIgG) served as negative control capture reagents. * = non-specific bands.

**Fig 4:**
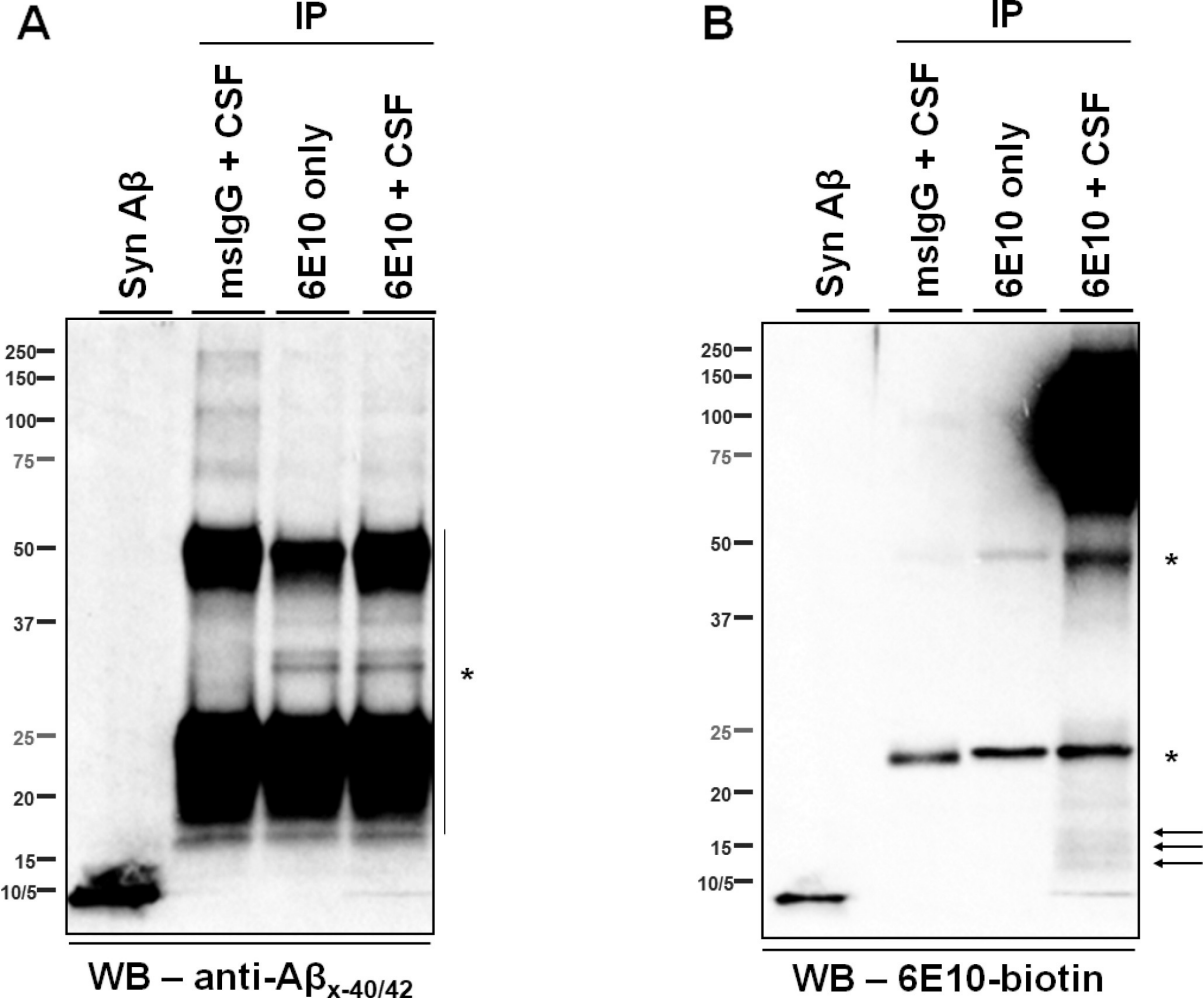
The C-termini of Aβ were not detected in the ~15-kDa, 6E10-immunoreactive proteins of human CSF. **(A)** The monoclonal antibody 6E10 was used to immunoprecipitate proteins from CSF, and WB was probed with C-terminal end-specific monoclonal antibodies targeting Aβ_40_ and Aβ_42_. No ~15-kDa proteins were detected. To serve as negative controls, mouse IgG replaced 6E10 as the capture reagent (msIgG + CSF) or 6E10 was incubated with immunoprecipitation dilution buffer in the absence of CSF (6E10 only). Syn Aβ, synthetic Aβ_1–40_ (5 ng) and Aβ_1–42_ (5 ng). **(B)** The same blot shown in (A) was stripped with Restore PLUS stripping buffer (Thermo Fisher Scientific) and reprobed with biotinylated 6E10, and the ~15-kDa proteins (arrows) were then detected. * = non-specific bands.

To investigate the composition of the ~55- and the ~15- kDa, 6E10-immunoreactive proteins, CSF was first immunoprecipitated using a series of monoclonal antibodies targeting the N-terminal region (a.a. 1–16), the mid-region (a.a.17-24), and the C-terminus (Aβ_x-40/42_) of Aβ (**Fig 1**); the eluates were then analyzed by WB using biotinylated 6E10 as the detection antibody. The ~55-kDa proteins were detected regardless of which antibody was used for IP (**Fig 3A**). Notably, bands from the 4G8 (targeting the mid-region of Aβ) IP were only visualized when blots were exposed for longer times, possibly because this epitope was partially masked during immunocapture. Indeed, the presence of Aβ fragments recognized by 4G8 in ~55-kDa proteins was confirmed by direct WB of CSF (**S2 Fig**). In addition, the ~55-kDa proteins were detected by the monoclonal antibody 82E1, which recognizes the N-terminus of a canonical Aβ (residue +1), in eluate from CSF immunoprecipitated with antibodies recognizing the C-termini of Aβ_40_ and Aβ_42_ (**Fig 3A**). These findings support the presence of canonical Aβ_1-40/42_ in the ~55-kDa, 6E10-immunoractive proteins. In contrast, the ~15-kDa proteins were detected by 6E10 only in eluates from CSF that had been immunoprecipitated with antibodies recognizing the N-terminal region of Aβ (*i*.*e*., 6E10 and 42.5, **Fig 3A**). To rule out the possibility that the C-terminal region of Aβ in the ~15-kDa proteins is present but inaccessible to the capture antibodies, CSF was immunoprecipitated by 6E10 and eluted proteins were revealed by antibodies against the C-termini of Aβ. We did not detect any ~15-kDa proteins under such circumstances, although they occurred when the same blots were re-probed by 6E10 (**Fig 4**). Further, direct WB of CSF samples probed with either 4G8 or anti-Aβ_x-40/42_ antibodies detected ~55- but no ~15- kDa proteins (**S2 Fig**). Taken together, these results indicate that the ~15-kDa, 6E10-immunoreactive proteins do not harbor the mid- or C-terminal region of Aβ.

**Fig 5:**
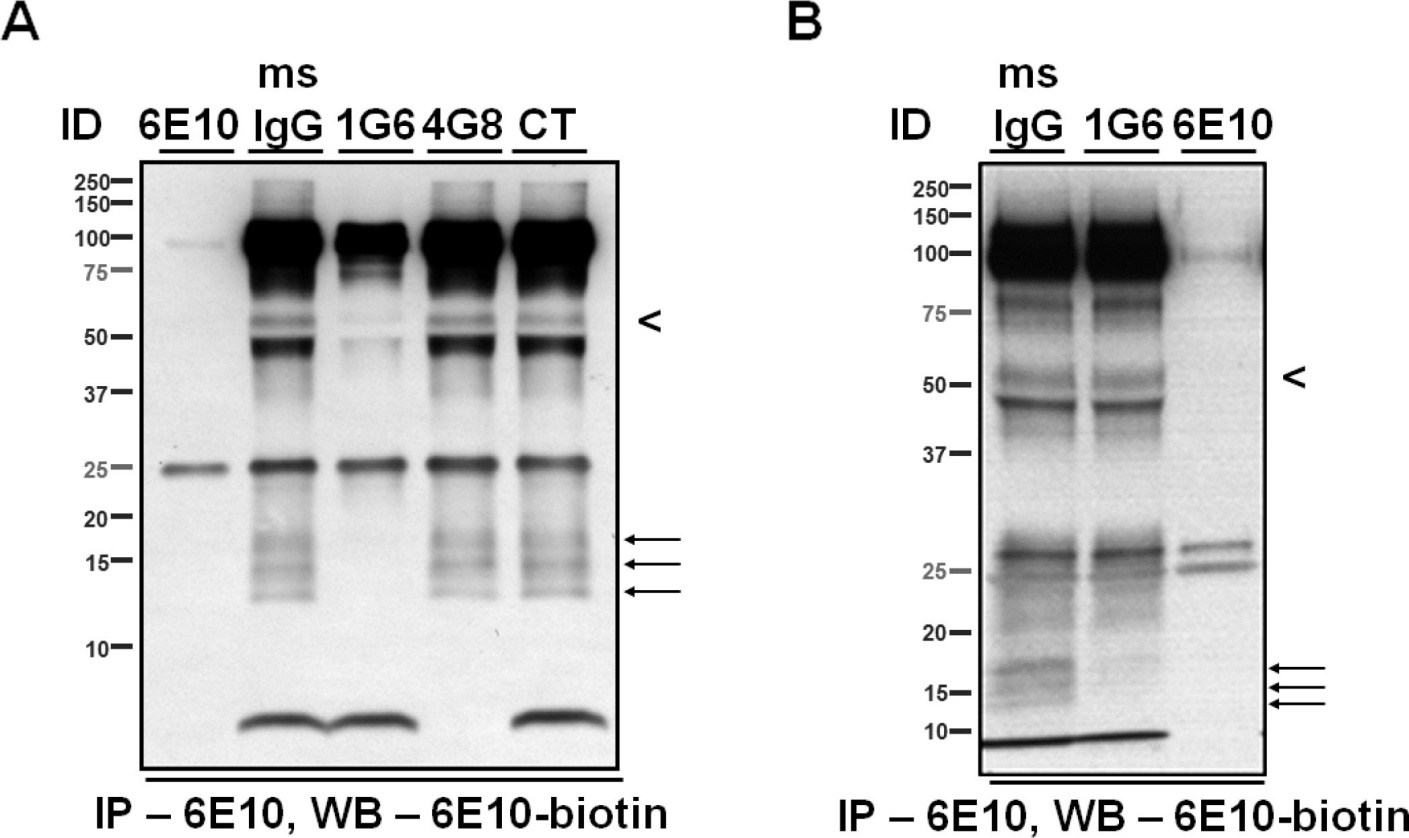
The ~55- and the ~15- kDa, 6E10-immunoreactive species contain APP metabolites located N-terminally of Aβ. **(A)** CSF samples were first immunodepleted using the antibodies noted above each lane, and then subjected to immunoprecipitation with 6E10, and WB was probed with biotinylated 6E10. Immunodepletion with 6E10 removed both the ~55- (arrowhead) and the ~15- kDa (arrows) proteins as expected. Immunodepletion with the 1G6 antibody fully removed the ~15-kDa species and partially removed the ~55-kDa species, indicating the presence of APP fragments located N-terminally of Aβ in these bands. In contrast, an antibody directed against the C-terminus (CT) of APP and the antibody 4G8 failed to deplete the ~55- and the ~15- kDa, 6E10-immunoreactive proteins. Note that the blot was transferred from a 10–20% Tris-Tricine gel. **(B)** In different CSF samples, immunodepletion with the antibody 1G6 did not reduce levels of the ~55-kDa band, but almost fully eliminated the ~15-kDa band. Immunodepletion with mouse IgG (msIgG) served as a negative control. Note that the blot was transferred from a 10.5–14% Tris-HCl gel.

Next, we determined whether the ~55- and the ~15- kDa, 6E10-immunoreactive proteins are comprised of APP metabolites other than Aβ. CSF samples were immunoprecipitated by antibodies against various regions of APP (**Fig 1**). The ~15-kDa proteins were detected by biotinylated 6E10 in eluates from CSF that had been immunoprecipitated using the monoclonal antibody 1G6, which is directed against APP_648-651_ (APP_770_ numbering system hereafter unless specified), but not when antibodies targeting the N-terminal (NT, APP_104-118_), or C-terminal (CT, APP_751-770_) region of APP were used (**Fig 3B**). The ~55-kDa, 6E10-immunoreactive proteins were also immunoprecipitated by 1G6, but not by the antibodies directed against N- or C-terminal regions of APP (**Fig 3B**). To rule out the possibility that the C-terminal regions of APP are present in the ~55- and/or the ~15- kDa proteins but inaccessible to the capture antibody, direct WB of CSF was performed using CT as the detection antibody. As a result, neither the ~55- nor the ~15- kDa proteins were detected (**S2 Fig**). Since NT only recognizes target proteins under non-denaturing conditions, no WB of CSF was performed using this antibody. Notably, the intensity of the ~55-kDa band from the 1G6 IP is lower than the intensity from the 6E10 IP, while the intensities of the ~15-kDa bands are similar from both IPs. These differences may reflect 1) differences in the structures of the ~55- and the ~15- kDa, 1G6-immunoreactive proteins that cause the 1G6 epitope to be less accessible in the ~55-kDa species, and/or 2) differences in the relative levels of 1G6-immunoreactive proteins contributing to the bands at ~55 kDa and ~15 kDa. To further confirm these results, CSF samples were first depleted of APP and/or Aβ using antibody 6E10, 1G6, 4G8 or CT, and then subjected to immunocapture by 6E10. The eluates were then detected using 6E10 (**Fig 5A**). The ~15-kDa proteins were no longer detected by 6E10 following pre-treatment of CSF by either 6E10 or 1G6 (**Fig 5**). Although the ~55-kDa proteins were fully depleted by 6E10 as expected, they were only partially (**Fig 5A**) or barely depleted by 1G6 (**Fig 5B**), consistent with the weakness of the signal in the 1G6 IP. These findings support the presence of APP metabolites located N-terminally of Aβ in the ~55- and the ~15- kDa, 6E10-immunoreactive proteins.

**Fig 6:**
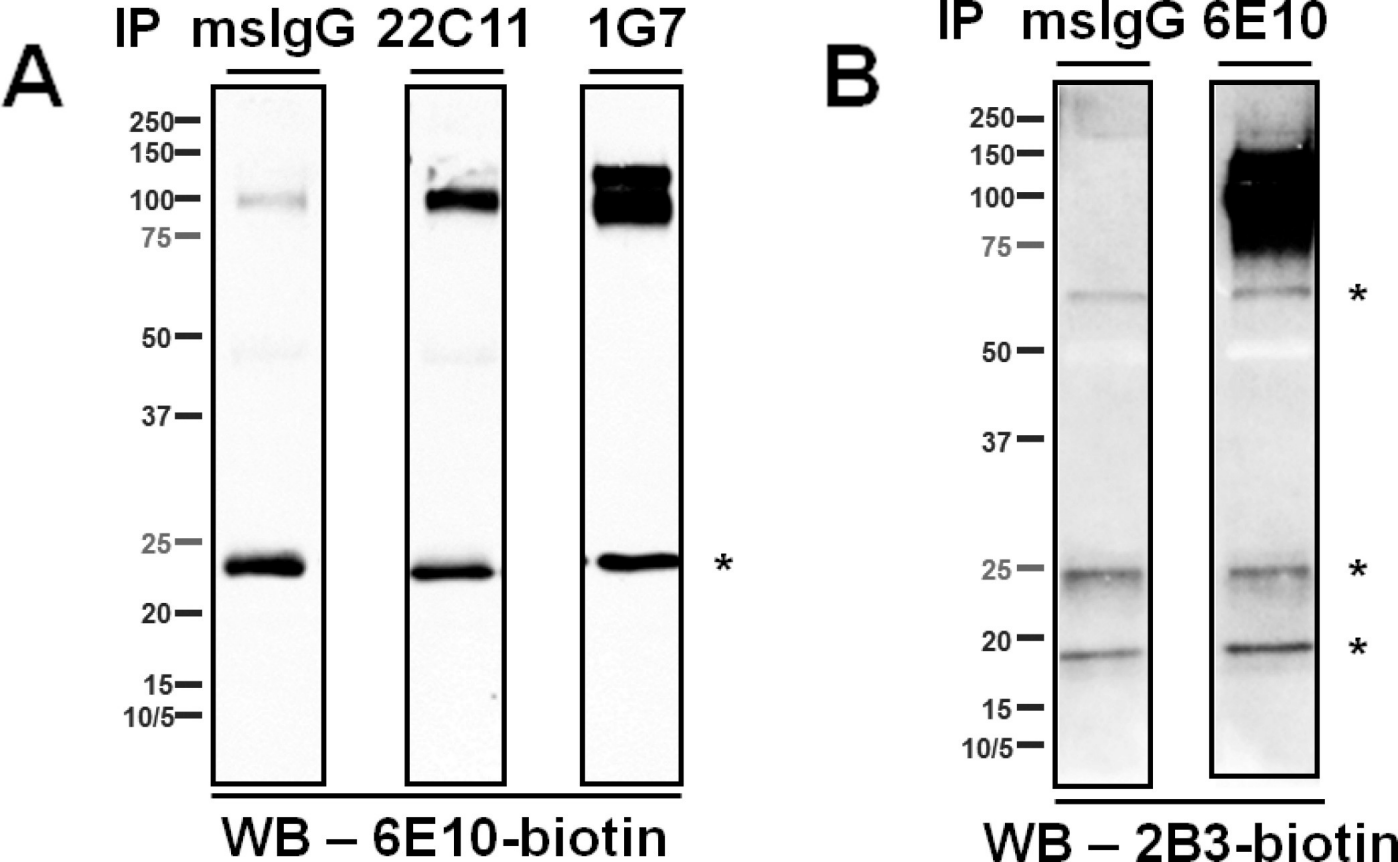
Mapping the N- and C-termini of the ~55- and the ~15- kDa, 6E10-immunoreactive proteins. **(A)** CSF samples were immunoprecipitated using either 22C11 or 1G7, directed against APP_66-81_ and APP_380-665_, respectively, and WB was probed with biotinylated 6E10. Neither antibody captured the ~55- or the ~15- kDa proteins. **(B)** CSF samples were immunoprecipitated using 6E10, and immunocaptured proteins were detected using biotinylated 2B3, directed against the α-secretase cleavage site of APP. Neither the ~55- nor the ~15- kDa proteins were detected. In both experiments, mouse IgG (msIgG) was used as a negative control capture reagent. * = non-specific bands.

To map the N-terminus of these APP metabolites, we immunoprecipitated CSF samples using the monoclonal antibodies 22C11 and 1G7 targeting APP_66-81_ and APP_380-665_, respectively (**Fig 6A**), in addition to the antibody NT targeting APP_104-118_ (**Fig 3B**). We detected neither the ~55- nor the ~15- kDa, 6E10-immunoreactive proteins. In contrast, all three antibodies captured the ~100-kDa, 6E10-immunoreactive proteins, which most likely include the soluble α-secretase cleavage product of APP (sAPPα). To rule out the possibility that the regions of APP that are recognized by 22C11 or 1G7 are present in the ~55- and/or the ~15- kDa proteins but inaccessible to the capture antibody, direct WB of CSF was performed using 22C11 or 1G7 as the detection antibodies. Neither the ~55- nor the ~15- kDa proteins were detected by 22C11 (**S2 Fig**); while both ~55- and ~15- kDa proteins were detected by 1G7 (**S2 Fig**), they may not (fully) represent the 6E10-immunoreactive protein species characterized in this study. To map the C-termini of these APP fragments, we used the monoclonal antibody 2B3 that specifically binds Aβ/APP species ending at amino acid 15 in the Aβ sequence [37] to detect 6E10-immunoprecipitated CSF proteins. We observed neither the ~55- nor the ~15- kDa proteins (**Fig 6B**) despite detection of the ~100-kDa proteins such as sAPPα, indicating that neither the ~55- nor the ~15- kDa bands contained species ending at the α-secretase cleavage site of APP.

### Mass spectrometry identified APP fragments in the ~55- and the ~15- kDa, 6E10-immunoreactive proteins in CSF

**Fig 7:**
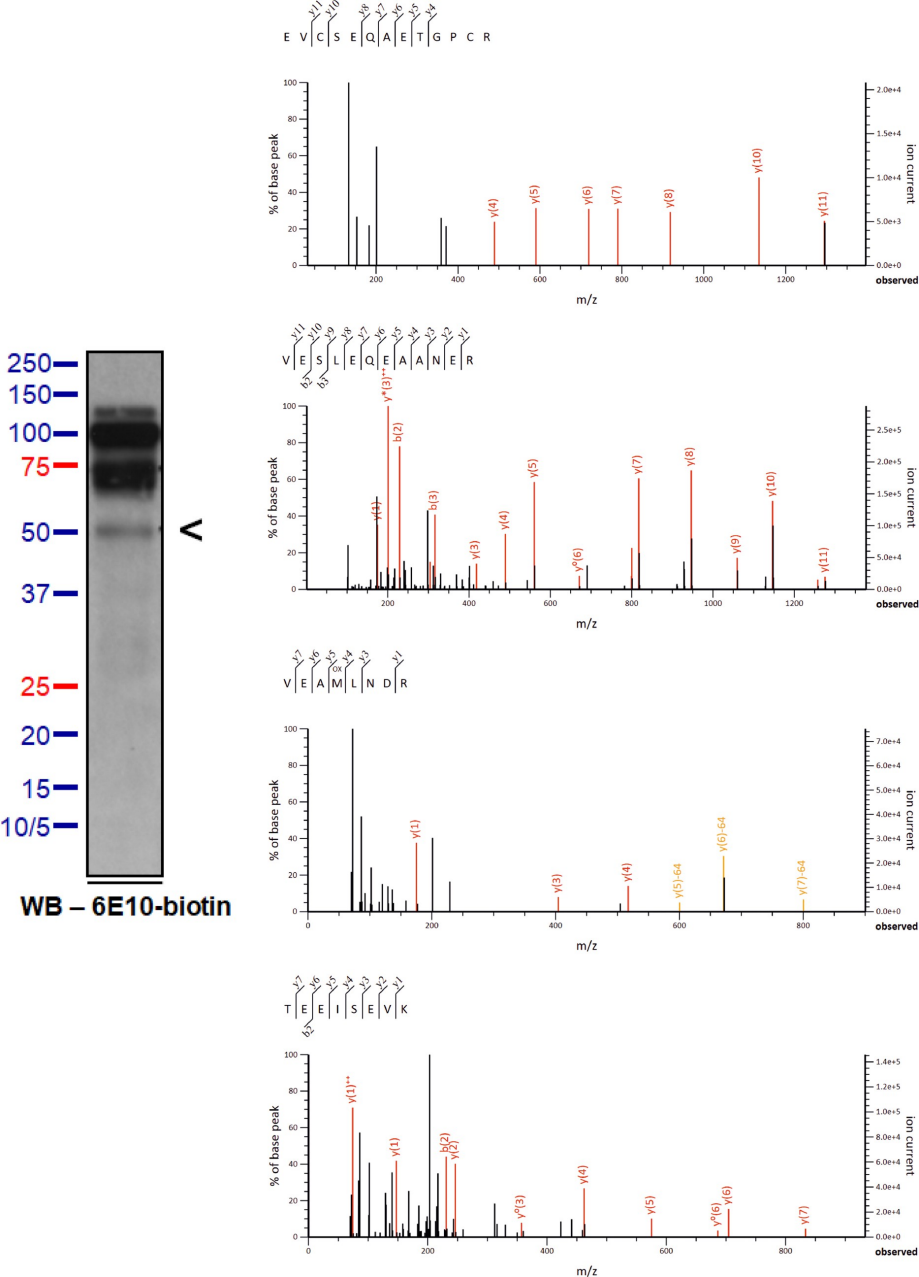
In-gel trypsin digestion/MS analysis of the ~55-kDa, 6E10-immunoreactive proteins of human CSF samples. The location of the ~55-kDa target proteins was identified in unstained gels by overlaying the analytic lanes on the film record of the Western blot of the reference lane (shown at left, an arrowhead pointing to the ~55-kDa, 6E10-immunoreactive species). After alignment by the molecular weight standards, the pieces of unstained gel overlaying the bands of interest were excised. The isolated bands were subjected to in-gel trypsin digestion followed by MS analysis. The MS/MS spectra of the identified peptide fragments are shown in the right panel.

**Fig 8:**
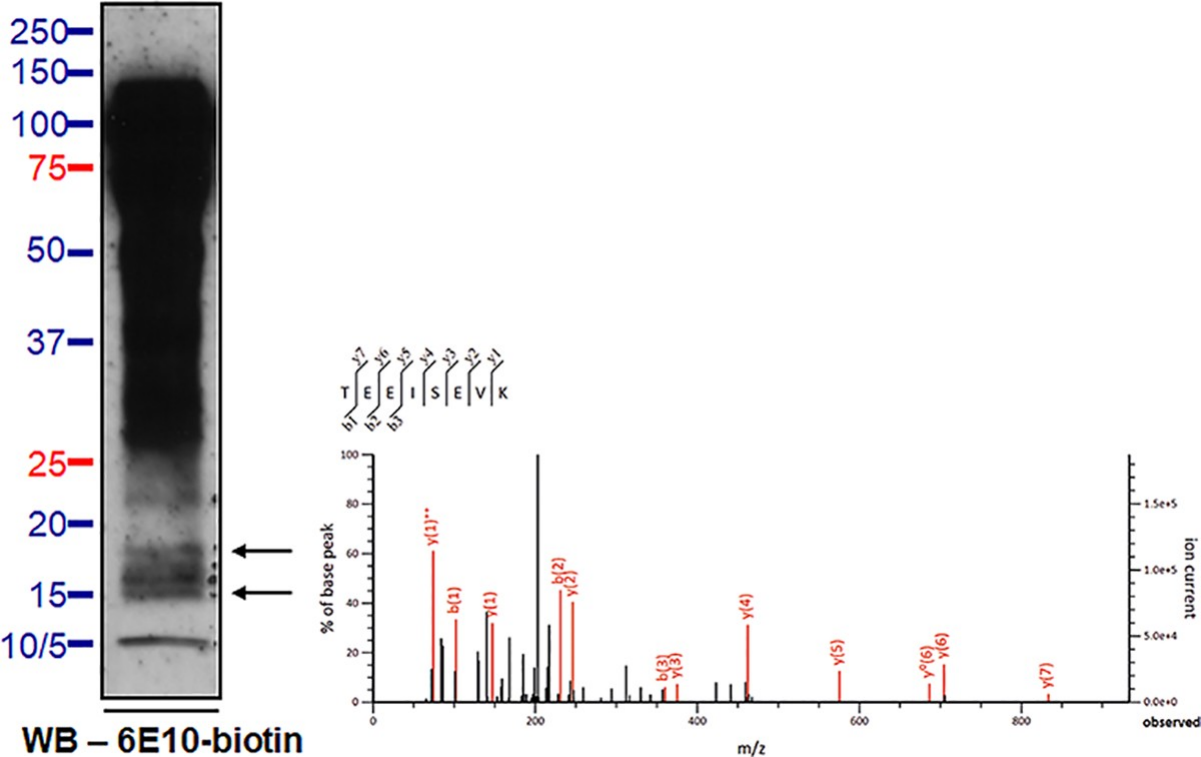
In-gel trypsin digestion/MS analysis of the ~15-kDa, 6E10-immunoreactive proteins of human CSF samples. The location of the ~15-kDa target proteins was identified in unstained gels by overlaying the analytic lanes on the film record of the Western blot of the reference lane (shown at left, arrows pointing to ~15-kDa, 6E10-immunoreactive bands from which peptides were identified by MS). After alignment by the molecular weight standards, the pieces of unstained gel overlaying the bands of interest were excised. The isolated bands were subjected to in-gel trypsin digestion followed by MS analysis. The MS/MS spectrum of the identified peptide fragment is shown in the right panel.

**Fig 9:**
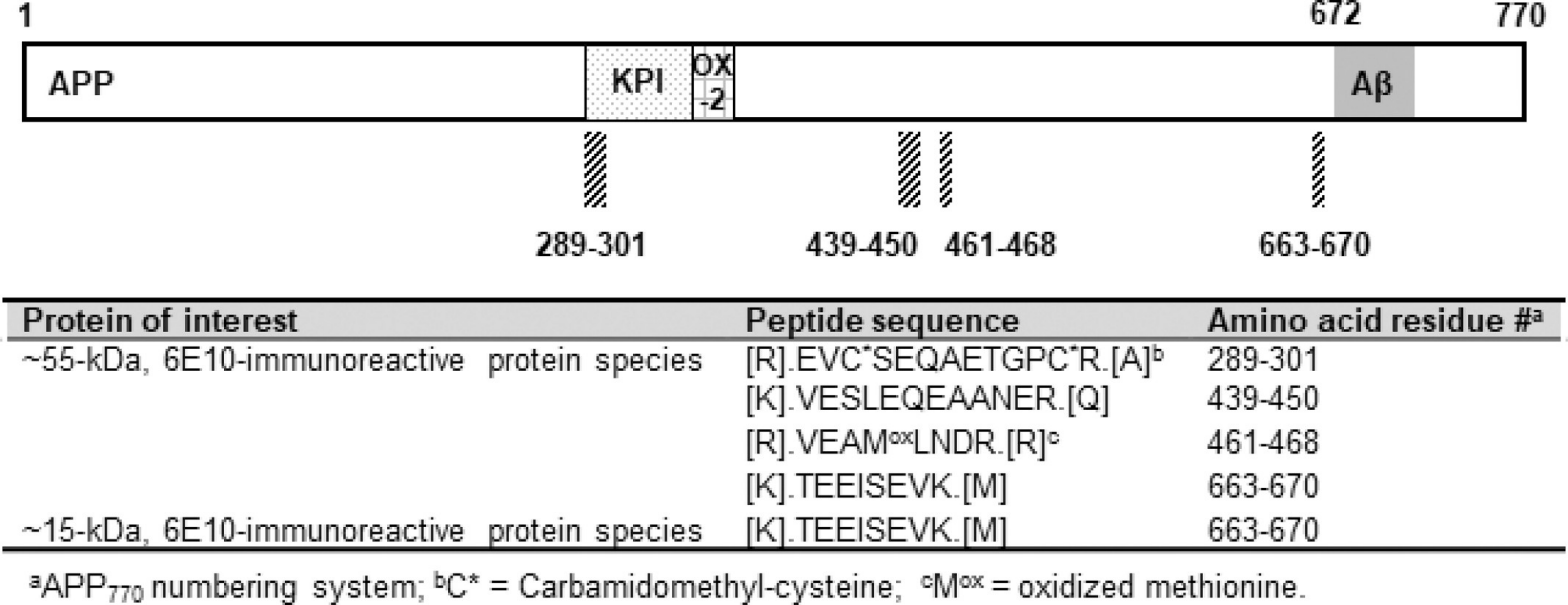
Aβ/APP fragments in the ~55- and the ~15- kDa, 6E10-immunoreactive proteins identified by mass spectrometry. Schematic diagrams showing peptide fragments (striated blocks) in the ~55- or the ~15- kDa species. The sequences of MS-identified peptide fragments are listed in the table below the diagram. KPI = the Kunitz-like protease inhibitor domain, present in the APP_770_ and APP_751_ isoforms but absent in the APP_695_ isoform; OX-2 = the OX-2 antigen domain, present in the APP_770_ isoform but absent in the APP_751_ and APP_695_ isoforms.

We used mass spectrometry (MS) to further explore the composition of the ~55- and the ~15- kDa, 6E10-immunoreactive proteins. CSF samples were immunoprecipitated by 6E10, and the antibody-bound proteins were separated by SDS-PAGE. For the ~55-kDa, 6E10-immunoreactive proteins, MS analysis identified several APP fragments that are located N-terminally of Aβ (*i*.*e*., APP_439-450_, APP_461-468_, and APP_663-670_) including a fragment (APP_289-301_) that partially overlapped with the N-terminal Kunitz-type protease inhibitor (KPI) domain (**Figs 7 and 9**). Notably, no APP fragments located C-terminally of Aβ were detected by MS, which is consistent with results of the IP/WB experiments. In addition, MS did not identify any mid- or C-terminal regions of Aβ (a.a. 17-x). For the ~15-kDa, 6E10-immunoreactive proteins, we identified fragments covering APP_663-670_ (*i*.*e*., -9Aβ-2) (**Figs 8 and 9**), which is consistent with findings from IP/WB. The results indicated that neither the mid-/C-terminal region of Aβ species nor the APP fragments located C-terminally of Aβ are present in these ~15-kDa proteins.

## Discussion

The goal of this study was to further characterize 6E10-immunoreactive species that we had previously reported to be elevated in the CSF of elderly individuals at risk for AD [23]. Using IP/WB coupled with IP/MS, we show that canonical Aβ_1-40/42_ peptides, together with APP fragments located N-terminally of Aβ, are found in the protein species that migrate at ~55 kDa. We also show that APP fragments located N-terminally of Aβ, plus the N-terminal region of Aβ, are present in the ~15-kDa species.

Our IP/WB data support the presence of canonical Aβ_1-40/42_ in the ~55-kDa, 6E10-immunoreactive band, although we cannot rule out the possibility that separate Aβ fragments from the N-terminal, mid-region, and C-terminal region form an SDS-stable complex autonomously or with other molecules. Although we did not detect Aβ by MS, this might be attributed to its low abundance in the ~55-kDa band; this failure to observe Aβ fragments is unlikely to be due to an inability of the MS experimental conditions to detect these fragments, since the mid-region of Aβ (*i*.*e*., Aβ_17–28_) was detected by MS in the <10-kDa, 6E10-immunoreactive CSF proteins (**S3 Fig**). The presence of intact canonical Aβ peptides in CSF has, nonetheless, been previously reported using IP/MS [38].

Using monoclonal antibodies targeting three distinct APP epitopes located N-terminally of Aβ, we failed to capture the ~55-kDa, 6E10-immunoreactive proteins. It is possible that the epitopes of 22C11 and NT (APP_66-81_ and APP_104-118_, respectively) are not present in these proteins since MS identified APP_289-301_ as the most N-terminal APP fragment. However, the failure to detect the ~55-kDa, 6E10-immunoreactive proteins by 1G7 IP may arise from the low abundance (compared to the ~100-kDa, 6E10-immunoreactive proteins) of the ~55-kDa proteins, and/or the inaccessibility of 1G7 epitope of the ~55-kDa proteins (not precisely mapped yet; Edward Koo, personal communications) to the antibody in non-denaturing conditions. The identification of APP_439-450_, APP_461-468,_ and APP_663-670_, together with APP_289-301_, a fragment partially overlapping with the N-terminal KPI domain of the APP_770_ and APP_751_ isoforms, indicates the complex composition of this band.

Of note, full-length KPI-containing APP isoforms and their soluble derivatives are present in human CSF [33, 39–41], and comprise a minor portion (approximately no more than 10%) of total CSF APP [42, 43]. Echoing this finding, we estimate that APP metabolites from the 751/770 isoforms together make up about 25% of the total CSF APP through selected reaction monitoring MS measurements. Previous biochemical characterization of the KPI-containing APP derivatives indicated that C-terminally truncated fragments with the N-terminus starting at amino acid residue 18 are present in CSF [39]. Quantitative analysis of these APP fragments identified an elevation in their levels of AD patients compared to non-demented subjects [33, 44–46], although contradictory findings have been reported [41, 43]. Nonetheless, the KPI-containing APP derivatives of CSF represent potential AD biomarkers. While these APP derivatives have apparent sizes of >100 kDa in Western blots, our results, for the first time, indicated that KPI-containing APP fragments constitute the ~55-kDa, 6E10-immunoreactive protein species that are present in lumbar CSF. Interestingly, using MS we detected specific peptides of APP_695_ from its recombinant form but not any single tryptic peptide that proves the presence of APP_695_ or APP_770_ in CSF, which may be due to unknown post-translational modifications. One such modifier may be *O*-glycosylation, whose presence may hinder the trypsinization or lead to our inability to find the peptides since we do not know the exact post-translational modifications to include in the database search. Together, these data lead us to suggest that the ~55-kDa, 6E10-immunoreactive band contains canonical Aβ_1-40/42_ as well as APP fragments N-terminal to Aβ, though the levels of the latter vary between different CSF samples. Meanwhile, these findings are consistent with a previous study showing the presence of N-terminal APP fragments and canonical Aβ in human CSF [31]. As a limitation of the study, we note that the aggregation state of these peptides is not yet clear, nor is it known whether non-Aβ/APP proteins are present in these complexes.

The ~15-kDa, 6E10-immunoreactive bands were immunodepleted by 1G6, indicating that these species extend N-terminal to Aβ, which is consistent with the IP/MS findings. We attempted to map the C-termini of the ~15-kDa bands using IP/WB with detection by the 2B3 antibody, but failed to identify signals. Interestingly, Aβ_1–15_ was previously detected by MS from 2B3-bound CSF proteins [37]; indeed, multiple peptides spanning both the 1G6 epitope and the β-secretase cleavage site and ending at glutamine-15 of Aβ have been previously identified in lumbar CSF samples [30, 32, 37]. Further, it is established that APP is also cleaved by other proteases, such as η-secretase [47], and δ-secretase (asparagine endopeptidase) [48]. Cleavage of APP by a combination of η- and α- secretase or a combination of δ- and α- secretase results in APP_580-687_ and APP_661-687_, respectively. The involvement of these APP fragments in comprising the ~15-kDa proteins warrants further investigation.

To understand the composition of the ~55- and the ~15- kDa, 6E10-immunoreactive proteins, we performed biochemical and mass spectrometry analyses on CSF specimens from both AD patients and non-AD individuals, and in some experiments, pooled AD and non-AD CSF samples. Although the studied protein species are generally present in lumbar CSF of both AD patients and normal individuals, potential variation in the composition of the ~55- and the ~15- kDa, 6E10-reactive protein species in the CSF of AD patients versus normal individuals likely exists. It is plausible that such variation, if present and consistently identified, may be used as a molecular tool for AD diagnosis, which represents one direction of our future investigation.

Overall, our findings suggest that Aβ aggregates combine with APP metabolites in human CSF specimens; they further highlight the importance of employing complementary experimental methods to determine the identities disease-relevant molecules of interest.

## Supporting information

S1 Fig Pilot Studies for localizing proteins of interest in unstained gels. (**A**) The blotting process did not distort the positions of proteins. Pre-stained protein standards were separated by electrophoresis on 10–20% Tris-Tricine or 10.15–14% Tris-HCl gels. The locations of the protein standards within the gels were manually recorded (G), then the proteins were electrophoretically transferred to nitrocellulose membranes, membranes were treated as for antigen-retrieval, and the locations of the proteins were again recorded (M). Numbers to the right show molecular weights (kDa). Note the alignment of the standards in the gels and the membranes. (**B**) Proteins can be localized in gels stored for 20 hr after SDS-PAGE. Western blots processed immediately after completion of SDS-PAGE (left, lanes 1–7) or after 20 hr storage of gel prior to electrophoretic transfer (right, lanes 8–12). Biotinylated 6E10 was used as the detection antibody. [Lane number] sample was shown above each lane. Lanes 1, 7, 8 and 12: pre-stained standards; lanes 2–5 and 10–11: equal amounts synthetic Aβ_1–40_ and Aβ_1–42_; lanes 6 and 9: proteins immunoprecipitated from cadaveric CSF using monocloncal antibody 6E10 (proteins from 250 μL of CSF each lane). (**C**) Co-localization of proteins in lanes processed immediately for WB or stored for 20 hr prior to transfer. i) lane 6 from blot shown in (B), processed immediately; ii) lane 9 from (B), processed after storage, flipped horizontally; iii) pseudocolor overlay of i (red) and ii (green), with locations of 37-50-kDa standards aligned; iv) pseudocolor overlay of i and ii, with locations of 10-20-kDa standards aligned. Short (10 sec) exposure. (**D**) Same series of lanes shown in (C), but longer exposure (30 sec) to better visualize band at ~50 kDa. (**E**) Protein alignment when unequal amounts of proteins loaded in lanes for immediate processing (i) and storage (ii). WB showing proteins immunoprecipitated from 250 μL (i) and 750 μL (ii) of cadaveric CSF; 6E10 for capture, biotinylated 6E10 for detection; (iii) pseudocolor overlay of i (red) and ii (green), with locations of 37-50-kDa standards aligned; (iv) pseudocolor overlay of i and ii, with locations of 10-20-kDa standards aligned.
(PDF)

S2 Fig WB of cerebrospinal fluid (CSF) samples with anti-APP/Aβ antibodies that were used for immunoprecipitation. Proteins of CSF samples were electrophoretically fractionated in SDS-PAGE, transferred onto nitrocellulose membranes, and probed with antibodies biotinylated 6E10 (**A-C**), 42.5 (**D**), 4G8 (**E**), anti-Aβ_x-40/42_ (**F**), biotinylated 1G6 (**G**), 1G7 (**H**), 22C11 (H) and CT (**I**). Although both ~55- (arrowhead) and ~15- kDa (arrows) proteins were detected using antibodies 6E10, 42.5, 1G6 and 1G7, the 42.5-, 1G6- and 1G7- reactive proteins may not (fully) represent the ~55- and ~15- kDa, 6E10-immunoreactive protein species characterized in this study; these proteins, unlike the 6E10-reactive, are thus highlighted by dash arrowhead and arrows. While 4G8 detected ~55- but no ~15- kDa proteins, 22C11 and CT detected neither protein species. In addition, since neither ~55- nor ~15- kDa proteins were detected by anti-Aβ_x-40/42_ (data not shown), we enriched proteins of interest by processing 500 μL of CSF sample (sample ID: 996) through size exclusion chromatography (F). We then detected ~55- but no ~15- kDa proteins reactive to anti-Aβ_x-40/42_. Vertical arrows indicate the fractions in which globular protein standards of the indicated molecular weights were eluted. The mismatch between the predicted elution fraction and molecular weights estimated by SDS-PAGE suggests that the anti-Aβ_x-40/42_-immunoreactive Aβ/APP metabolites do not migrate through the column as globular proteins. As negative controls, membranes were also probed using mouse immunoglobulin G (msIgG) for antibodies 42.5, 4G8, 1G7 and 22C11, rabbit immunoglobulin G (rbtIgG) for antibodies Anti-Aβx-40/42 and CT, or NeutrAvidin-horseradish peroxidase (HRP) for antibodies biotinylated 6E10 and biotinylated 1G6. Note: in Figure F, fraction volume: 250 μL, 50% was used for the anti-Aβ_x-40/42_ WB; In = 25 μL CSF sample.
(PDF)

S3 Fig In-gel trypsin digestion/MS analysis of the <10-kDa, 6E10-immunoreactive proteins of human CSF samples. The <10-kDa, 6E10-immunoreactive species (arrow) were identified in unstained gels by overlaying the analytic lanes on the film record of the Western blot of the reference lane, using the molecular weight standards for alignment; the pieces of unstained gel overlaying the bands of interest were excised. The isolated bands were subjected to in-gel trypsin digestion followed by MS analysis. The MS/MS spectrum of the identified peptide fragment is shown in the lower panel.
(PDF)

S1 Table Demographic characteristics of lumbar cerebrospinal fluid (CSF) providers. (DOCX)

S2 Table CSF sample information. (DOCX)
