# Supplementary figures and images for: Human cerebrospinal fluid 6E10-immunoreactive protein species contain amyloid precursor protein fragments
Source: PLoS One. 2019 Feb 28;14(2):e0212815. doi: 10.1371/journal.pone.0212815 (PMC6394962; doi:10.1371/journal.pone.0212815)

**A**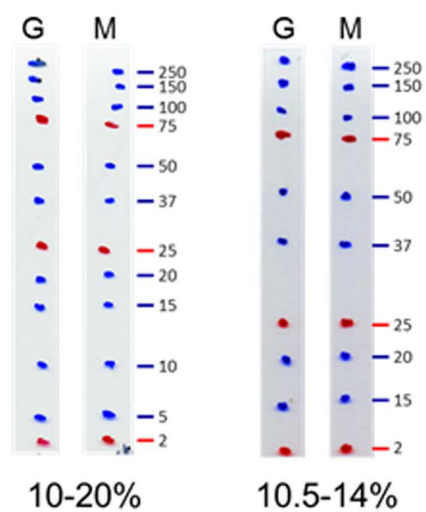**B**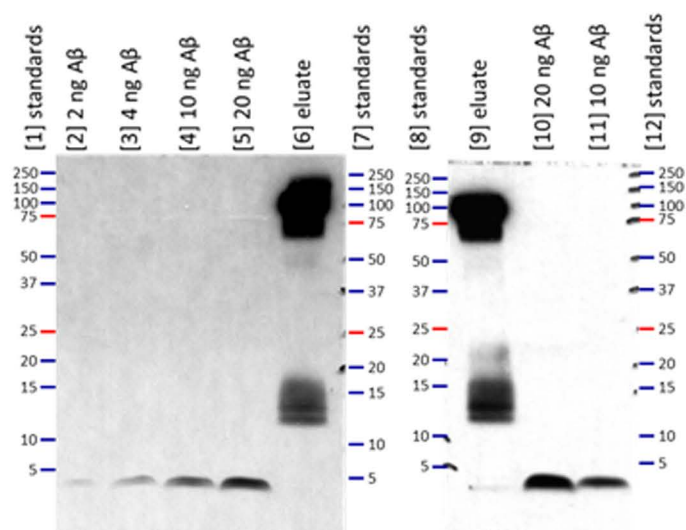**C**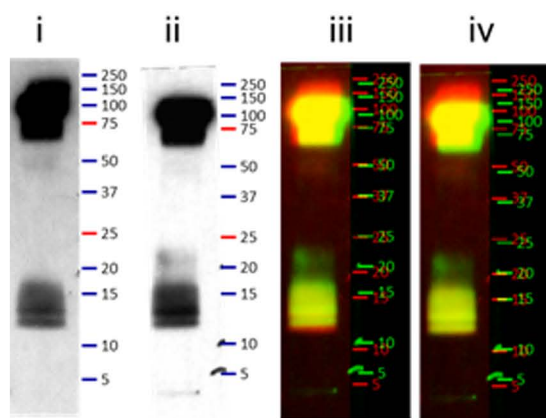**D**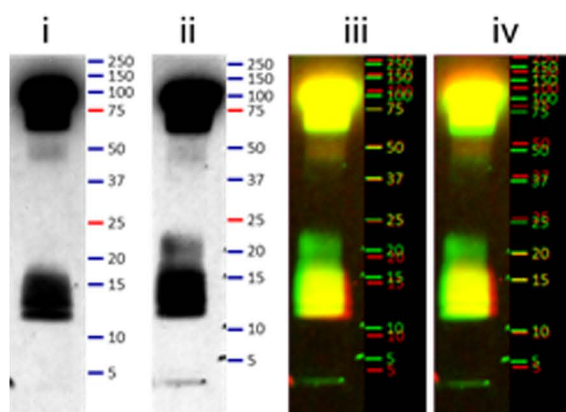**E**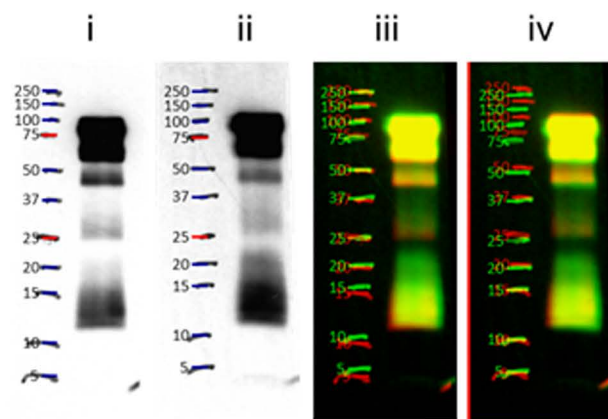

Supplement: S1 Fig — (A) The blotting process did not distort the positions of proteins. Pre-stained protein standards were separated by electrophoresis on 10–20% Tris-Tricine or 10.15–14% Tris-HCl gels. The locations of the protein standards within the gels were manually recorded (G), then the proteins were electrophoretically transferred to nitrocellulose membranes, membranes were treated as for antigen-retrieval, and the locations of the proteins were again recorded (M). Numbers to the right show molecular weights (kDa). Note the alignment of the standards in the gels and the membranes. (B) Proteins can be localized in gels stored for 20 hr after SDS-PAGE. Western blots processed immediately after completion of SDS-PAGE (left, lanes 1–7) or after 20 hr storage of gel prior to electrophoretic transfer (right, lanes 8–12). Biotinylated 6E10 was used as the detection antibody. [Lane number] sample was shown above each lane. Lanes 1, 7, 8 and 12: pre-stained standards; lanes 2–5 and 10–11: equal amounts synthetic Aβ1–40 and Aβ1–42; lanes 6 and 9: proteins immunoprecipitated from cadaveric CSF using monocloncal antibody 6E10 (proteins from 250 μL of CSF each lane). (C) Co-localization of proteins in lanes processed immediately for WB or stored for 20 hr prior to transfer. i) lane 6 from blot shown in (B), processed immediately; ii) lane 9 from (B), processed after storage, flipped horizontally; iii) pseudocolor overlay of i (red) and ii (green), with locations of 37-50-kDa standards aligned; iv) pseudocolor overlay of i and ii, with locations of 10-20-kDa standards aligned. Short (10 sec) exposure. (D) Same series of lanes shown in (C), but longer exposure (30 sec) to better visualize band at ~50 kDa. (E) Protein alignment when unequal amounts of proteins loaded in lanes for immediate processing (i) and storage (ii). WB showing proteins immunoprecipitated from 250 μL (i) and 750 μL (ii) of cadaveric CSF; 6E10 for capture, biotinylated 6E10 for detection; (iii) pseudocolor overlay of i (re [file pone.0212815.s001.pdf]

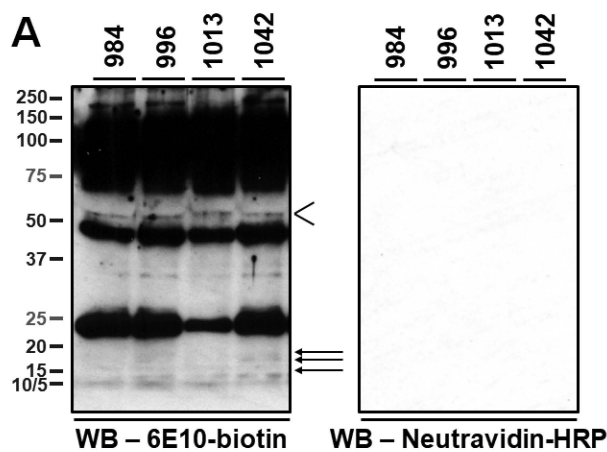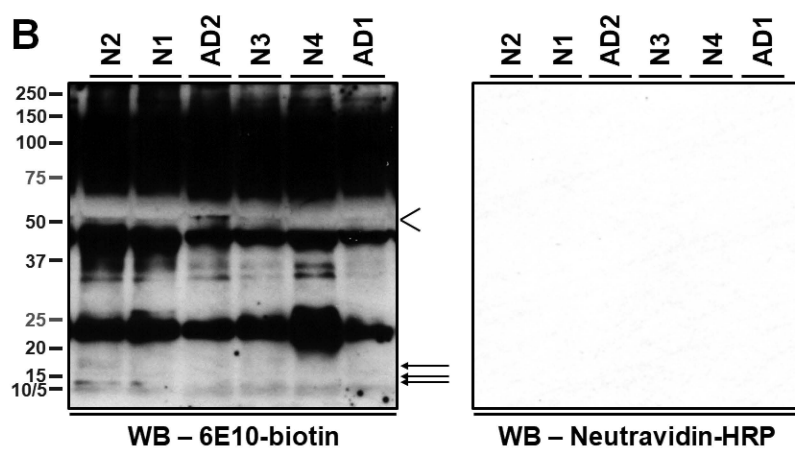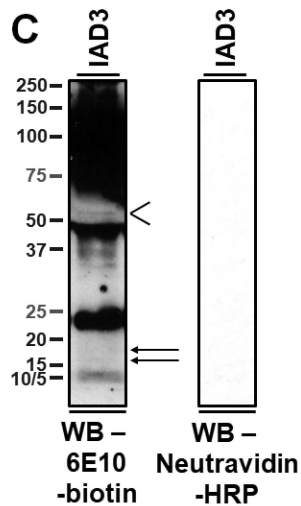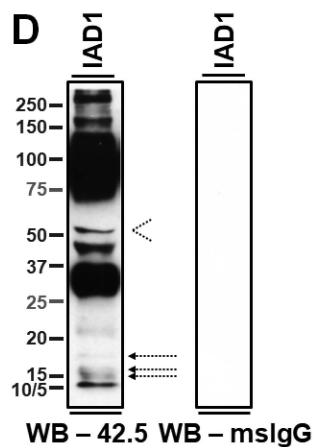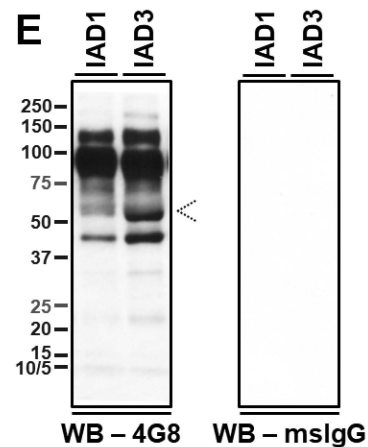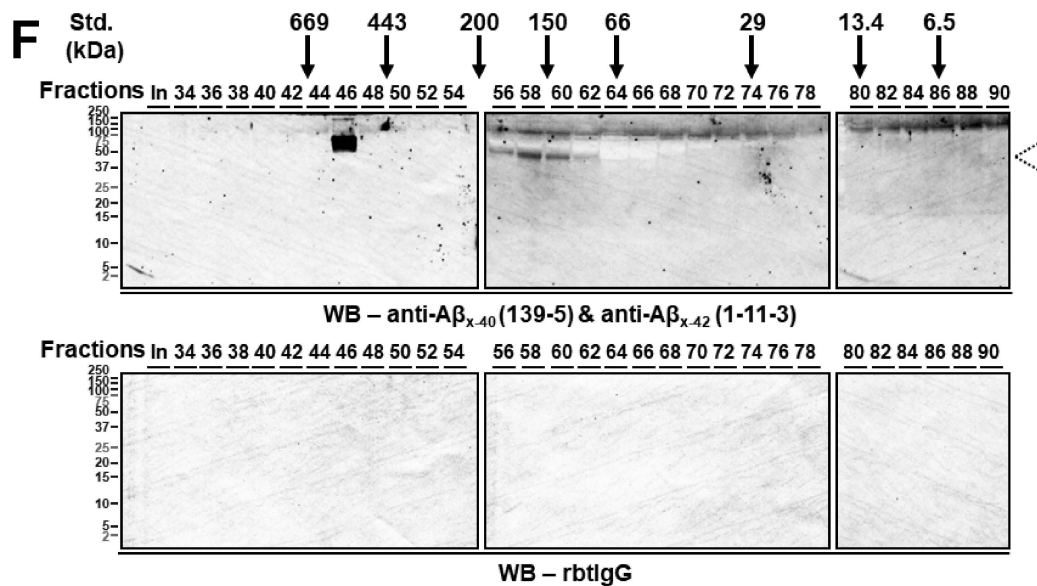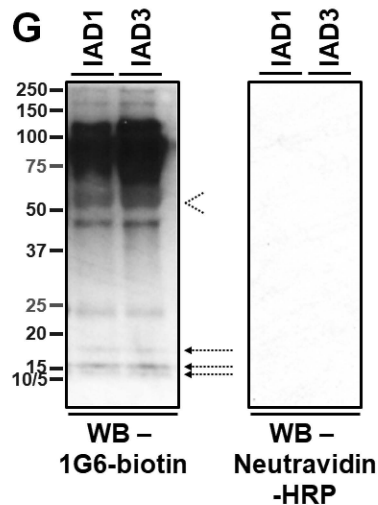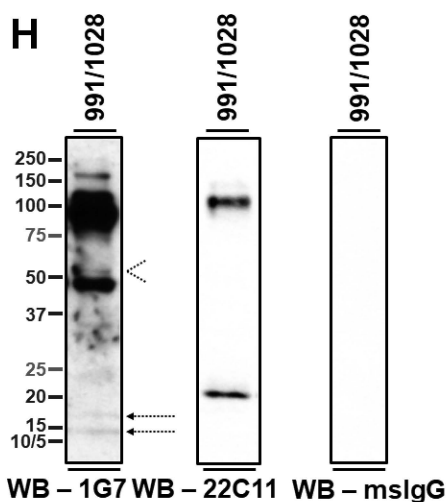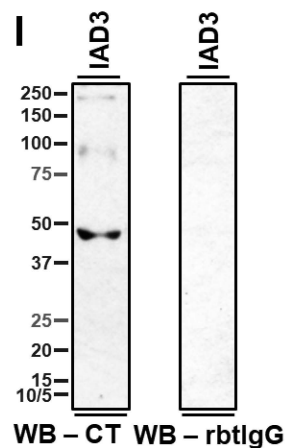

Supplement: S2 Fig — Proteins of CSF samples were electrophoretically fractionated in SDS-PAGE, transferred onto nitrocellulose membranes, and probed with antibodies biotinylated 6E10 (A-C), 42.5 (D), 4G8 (E), anti-Aβx-40/42 (F), biotinylated 1G6 (G), 1G7 (H), 22C11 (H) and CT (I). Although both ~55- (arrowhead) and ~15- kDa (arrows) proteins were detected using antibodies 6E10, 42.5, 1G6 and 1G7, the 42.5-, 1G6- and 1G7- reactive proteins may not (fully) represent the ~55- and ~15- kDa, 6E10-immunoreactive protein species characterized in this study; these proteins, unlike the 6E10-reactive, are thus highlighted by dash arrowhead and arrows. While 4G8 detected ~55- but no ~15- kDa proteins, 22C11 and CT detected neither protein species. In addition, since neither ~55- nor ~15- kDa proteins were detected by anti-Aβx-40/42 (data not shown), we enriched proteins of interest by processing 500 μL of CSF sample (sample ID: 996) through size exclusion chromatography (F). We then detected ~55- but no ~15- kDa proteins reactive to anti-Aβx-40/42. Vertical arrows indicate the fractions in which globular protein standards of the indicated molecular weights were eluted. The mismatch between the predicted elution fraction and molecular weights estimated by SDS-PAGE suggests that the anti-Aβx-40/42-immunoreactive Aβ/APP metabolites do not migrate through the column as globular proteins. As negative controls, membranes were also probed using mouse immunoglobulin G (msIgG) for antibodies 42.5, 4G8, 1G7 and 22C11, rabbit immunoglobulin G (rbtIgG) for antibodies Anti-Aβx-40/42 and CT, or NeutrAvidin-horseradish peroxidase (HRP) for antibodies biotinylated 6E10 and biotinylated 1G6. Note: in Figure F, fraction volume: 250 μL, 50% was used for the anti-Aβx-40/42 WB; In = 25 μL CSF sample. (PDF) [file pone.0212815.s002.pdf]

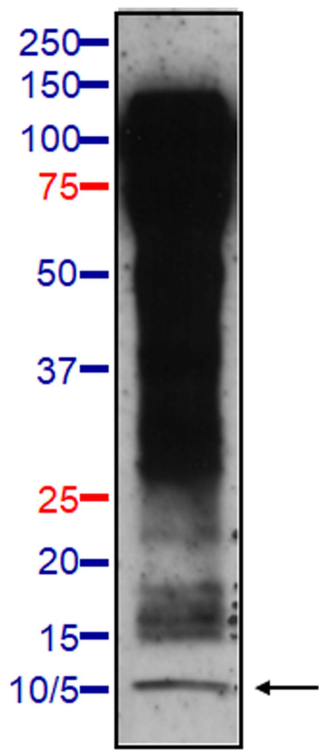

**WB – 6E10-biotin**

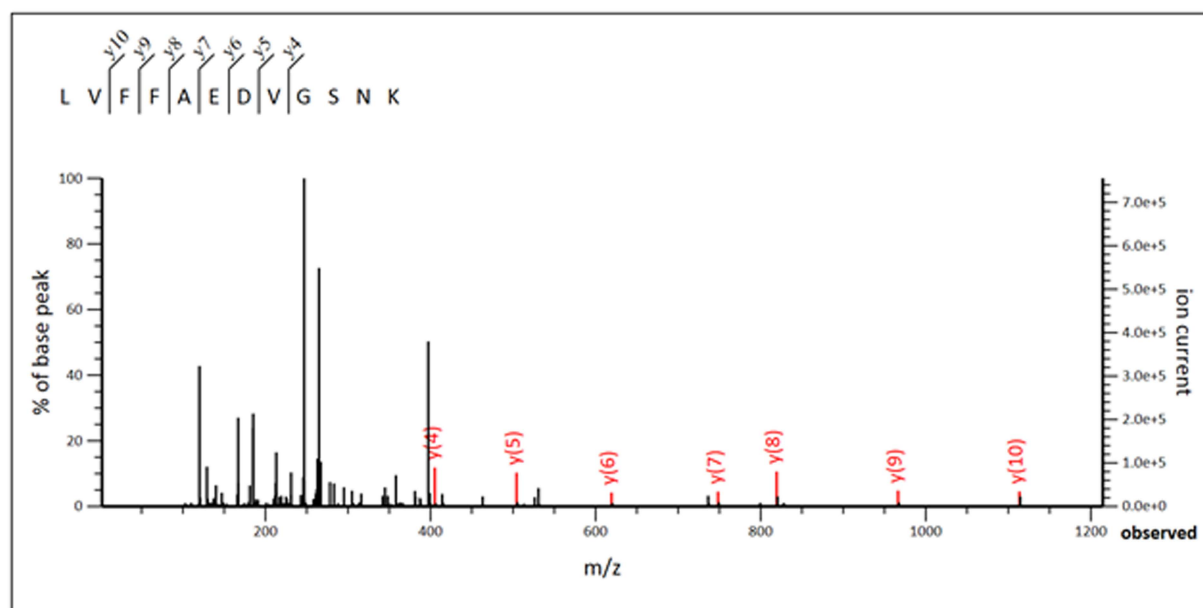

Supplement: S3 Fig — The <10-kDa, 6E10-immunoreactive species (arrow) were identified in unstained gels by overlaying the analytic lanes on the film record of the Western blot of the reference lane, using the molecular weight standards for alignment; the pieces of unstained gel overlaying the bands of interest were excised. The isolated bands were subjected to in-gel trypsin digestion followed by MS analysis. The MS/MS spectrum of the identified peptide fragment is shown in the lower panel. (PDF) [file pone.0212815.s003.pdf]
