# Supplementary material for: Human cerebrospinal fluid 6E10-immunoreactive protein species contain amyloid precursor protein fragments
Source: PLoS One. 2019 Feb 28;14(2):e0212815. doi: 10.1371/journal.pone.0212815 (PMC6394962; doi:10.1371/journal.pone.0212815)
Supplement: S1 Table — (DOCX) [file pone.0212815.s004.docx]

**S1 Table Demographic characteristics of lumbar cerebrospinal fluid (CSF) providers**

| **Sample ID** | **Age (yr)^a^** | **Sex^b^** | **Biomarker^c^** |
| --- | --- | --- | --- |
| 984 | 78 | M | Normal |
| 991 | 49 | F | Normal |
| 996 | 68 | M | Normal |
| 997 | 83 | F | Normal |
| 1013 | 72 | F | AD |
| 1028 | 71 | F | AD |
| 1041 | 77 | M | AD |
| 1042 | 81 | M | AD |

^a^Age at lumbar puncture cerebrospinal fluid (CSF) collection; ^b^M = male, F = female; ^c^CSF Aβ_42_ <400 pg/mL, AD; >550 pg/mL, Normal.

Note: The rest lumbar CSF samples (*i.e.*, N1, N2, N3, N4, AD1, AD2, lAD1 and lAD3) were pooled left-over aliquots of CSF samples whose providers were de-identified in clinical routine; demographic characteristics of the CSF providers were no longer traceable back to individuals.
