# Supplementary material for: Human cerebrospinal fluid 6E10-immunoreactive protein species contain amyloid precursor protein fragments
Source: PLoS One. 2019 Feb 28;14(2):e0212815. doi: 10.1371/journal.pone.0212815 (PMC6394962; doi:10.1371/journal.pone.0212815)
Supplement: S2 Table — (DOCX) [file pone.0212815.s005.docx]

**S2 Table CSF sample information**

| **Figures** | **Sample ID** | **Biomarker (CSF Aβ_42_: <400 pg/mL, AD; >550 pg/mL, Normal)** | **Sample size (*N*)** |
| --- | --- | --- | --- |
| 2A | 984, 991, 996, 997, 1013, 1028, 1041 and 1042 (lanes 1-8, left to right in this order) | 984, 991, 996 and 997, Normal; 1013, 1028, 1041 and 1042, AD | 8 (individual) |
| 2B | N3, N1, AD1, N4, AD2 and N2 (lanes 1-6, left to right in this order) | N1, N2, N3 and N4, Normal; AD1 and AD2, AD | 6 (each pooled from 2 subjects) |
| 2C | lAD3 | AD | 1 (pooled from 2-3 subjects) |
| 2D | 996 | Normal | 1 (individual) |
| 3A, left panel | lAD1 | AD | 1 (pooled from 2-3 subjects) |
| 3A, middle panel | 991 and 1028, pooled | 991, Normal; 1028, AD | 1 (pooled from 2 subjects) |
| 3A, right panel | 991 and 1028, pooled | 991, Normal; 1028, AD | 1 (pooled from 2 subjects) |
| 3B | lAD3 | AD | 1 (pooled from 2-3 subjects) |
| 4 | 996 | Normal | 1 (individual) |
| 5A | lAD3 | AD | 1 (pooled from 2-3 subjects) |
| 5B | lAD1 | AD | 1 (pooled from 2-3 subjects) |
| 6 | 991 and 1028, pooled | 991, Normal; 1028, AD | 1 (pooled from 2 subjects) |
| 7 | 991, 997, 1028 and 1041, pooled | 991 and 997, Normal; 1028 and 1041, AD | 1 (pooled from 4 subjects) |
| 8 | 991, 997, 1028 and 1041, pooled | 991 and 997, Normal; 1028 and 1041, AD | 1 (pooled from 4 subjects) |
| S1 | Cadaveric CSF samples (ID, not known) | Not known | 1 (individual) |
| S2A | 984, 996, 1013 and 1042 (lanes 1-4, left to right in this order) | 984 and 996, Normal; 1013 and 1042, AD | 4 (individual) |
| S2B | N2, N1, AD2, N3, N4 and AD1 (lanes 1-6, left to right in this order) | N1, N2, N3 and N4, Normal; AD1 and AD2, AD | 6 (each pooled from 2 subjects) |
| S2C | lAD3 | AD | 1 (pooled from 2-3 subjects) |
| S2D | lAD1 | AD | 1 (pooled from 2-3 subjects) |
| S2E | lAD1 and lAD3 | AD | 2 (each pooled from 2-3 subjects) |
| S2F | 996 | Normal | 1 (individual) |
| S2G | lAD1 and lAD3 | AD | 2 (each pooled from 2-3 subjects) |
| S2H | 991 and 1028, pooled | 991, Normal; 1028, AD | 1 (pooled from 2 subjects) |
| S2I | lAD3 | AD | 1 (pooled from 2-3 subjects) |
| S3 | 991, 997, 1028 and 1041, pooled | 991 and 997, Normal; 1028 and 1041, AD | 1 (pooled from 4 subjects) |
